# Stabilized recombinant SARS-CoV-2 spike antigen enhances vaccine immunogenicity and protective capacity

**DOI:** 10.1172/JCI159895

**Published:** 2022-12-15

**Authors:** Christian Meyer zu Natrup, Alina Tscherne, Christine Dahlke, Malgorzata Ciurkiewicz, Dai-Lun Shin, Anahita Fathi, Cornelius Rohde, Georgia Kalodimou, Sandro Halwe, Leonard Limpinsel, Jan H. Schwarz, Martha Klug, Meral Esen, Nicole Schneiderhan-Marra, Alex Dulovic, Alexandra Kupke, Katrin Brosinski, Sabrina Clever, Lisa-Marie Schünemann, Georg Beythien, Federico Armando, Leonie Mayer, Marie L. Weskamm, Sylvia Jany, Astrid Freudenstein, Tamara Tuchel, Wolfgang Baumgärtner, Peter Kremsner, Rolf Fendel, Marylyn M. Addo, Stephan Becker, Gerd Sutter, Asisa Volz

**Affiliations:** 1 Institute of Virology, University of Veterinary Medicine Hannover, Foundation, Hanover, Germany.; 2 Division of Virology, Department of Veterinary Sciences, LMU Munich, Munich, Germany.; 3 German Center for Infection Research, partner site Munich, and; 4 partner site Hamburg-Lübeck-Borstel-Riems.; 5 University Medical Center Hamburg-Eppendorf, Institute for Infection Research and Vaccine Development (IIRVD), Hamburg, Germany.; 6 Department of Pathology, University of Veterinary Medicine Hannover, Foundation, Hanover, Germany.; 7 University Medical Center Hamburg-Eppendorf, Division of Infectious Diseases, Hamburg, Germany.; 8 German Center for Infection Research, partner site Gießen-Marburg-Langen.; 9 Institute of Virology, Philipps University Marburg, Marburg, Germany.; 10 German Center for Infection Research, partner site Tübingen.; 11 Institute of Tropical Medicine, University of Tübingen, Tübingen, Germany.; 12 NMI Natural and Medical Sciences Institute at the University of Tübingen, Reutlingen, Germany.; 13 Centre de Recherches Médicales de Lambarene, Gabon.; 14 German Center for Infection Research, partner site Hanover-Braunschweig.

**Keywords:** Infectious disease, Vaccines, Adaptive immunity, Immunoglobulins, Molecular biology

## Abstract

The SARS-CoV-2 spike (S) glycoprotein is synthesized as a large precursor protein and must be activated by proteolytic cleavage into S1 and S2. A recombinant modified vaccinia virus Ankara (MVA) expressing native, full-length S protein (MVA-SARS-2-S) is currently under investigation as a candidate vaccine in phase I clinical studies. Initial results from immunogenicity monitoring revealed induction of S-specific antibodies binding to S2, but low-level antibody responses to the S1 domain. Follow-up investigations of native S antigen synthesis in MVA-SARS-2-S–infected cells revealed limited levels of S1 protein on the cell surface. In contrast, we found superior S1 cell surface presentation upon infection with a recombinant MVA expressing a stabilized version of SARS-CoV-2 S protein with an inactivated S1/S2 cleavage site and K986P and V987P mutations (MVA-SARS-2-ST). When comparing immunogenicity of MVA vector vaccines, mice vaccinated with MVA-SARS-2-ST mounted substantial levels of broadly reactive anti-S antibodies that effectively neutralized different SARS-CoV-2 variants. Importantly, intramuscular MVA-SARS-2-ST immunization of hamsters and mice resulted in potent immune responses upon challenge infection and protected from disease and severe lung pathology. Our results suggest that MVA-SARS-2-ST represents an improved clinical candidate vaccine and that the presence of plasma membrane–bound S1 is highly beneficial to induce protective antibody levels.

## Graphical abstract

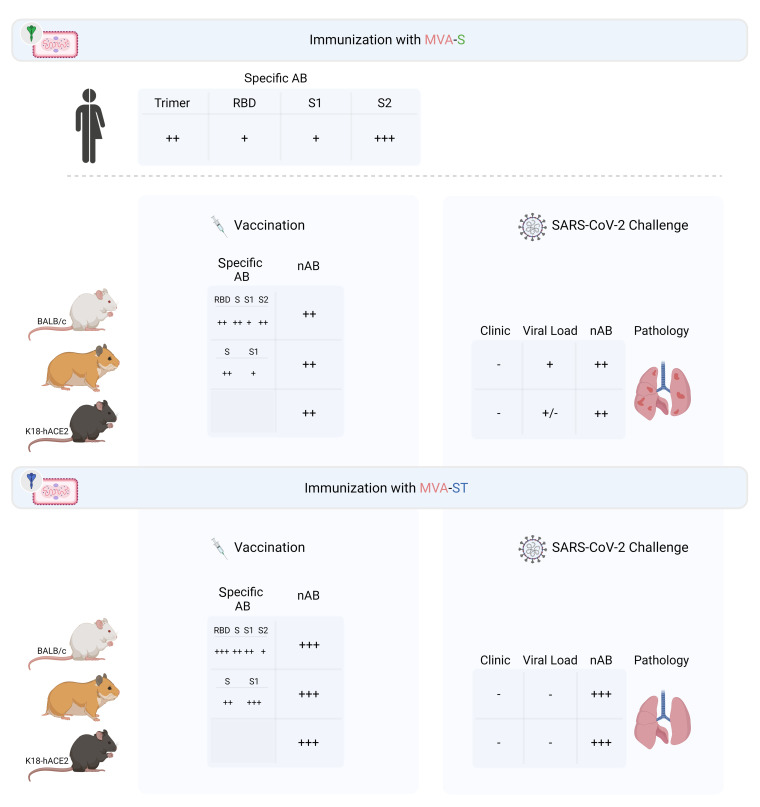

## Introduction

All COVID-19 vaccines licensed to date include the complete SARS-CoV-2 spike (S) protein as key antigen to elicit protective immune responses. Trimers of this large viral surface protein form the distinctive spikes of the coronavirus (1). Monomeric S is a glycosylated transmembrane protein consisting of a large N-terminal ectodomain and a short C-terminal endodomain. The full-length SARS-CoV-2 S protein is cleaved by a furin-like protease into 2 almost equally sized polypeptides called S1 (N-terminus of S) and S2 (membrane-anchored C-terminus of S). S1 harbors the receptor binding domain (RBD), which interacts with the cellular receptor molecule angiotensin-converting enzyme 2 (ACE2) and serves, together with other parts of S1, as an important target for antibodies that can interfere with host cell receptor binding capable of neutralizing SARS-CoV-2 infection. S2 mediates fusion between the virus and cell membrane, and is also an important target for antibodies that can interfere with virus entry.

S-specific virus-neutralizing antibodies are a major component of the vaccine-induced immune response protecting against SARS-CoV-2 infection (2). COVID-19 vaccines with reported efficacy deliver as an antigen either native S polypeptides (3–5) or modified versions of the full-length S protein (6–9). The modified S antigens contain 2 proline amino acid substitutions in the S2 protein between the fusion peptide and the first hinge region sequence to arrest the S protein in the prefusion conformation (1). Two S vaccine antigens harbor additional mutations to prevent S1/S2 cleavage by furin-like proteases (6, 8). While all of the different candidate vaccines based on S antigens elicit protective immunity in humans, they seem to induce distinct levels of vaccine efficacy and S-specific antibody responses (10). Structural features of the various S antigens might account for these differences in vaccine immunogenicity and/or vaccine efficacy and warrant further investigation. Moreover, recent studies demonstrated that the persistence of immune responses induced by approved COVID-19 vaccines and/or infection is limited. While all approved vaccine candidates provide a high level of protection against severe disease and death, protection against SARS-CoV-2 infection and/or transmission declines due to the waning of S-specific antibodies and the emergence of variants. To address this limitation, improved vaccination strategies that could be used as booster vaccines are urgently needed.

Modified vaccinia virus Ankara (MVA), a replication-deficient orthopoxvirus vaccine strain, has long served as an advanced vaccine technology platform for developing viral vector vaccines against emerging infectious disease (10–14).

Recent work addressed the preclinical development of MVA vector vaccines against COVID-19, including our candidate vaccine MVA-SARS-2-S (MVA-S) (15). Immunizations with MVA-S in animal models demonstrated the safety, immunogenicity, and protective efficacy of this vector vaccine delivering the native full-length SARS-CoV-2 S antigen. Further, MVA-S entered phase Ia clinical evaluation to assess the clinical safety and tolerability of 2 administrations and 2 ascending dose levels in healthy adults (ClinicalTrials.gov NCT04569383).

One objective of this study was to more closely examine the S-specific antibody responses following MVA-S immunization. Preliminary data from this immunogenicity monitoring suggested that most of the vaccine-induced native S–antigen-specific antibodies bound to the S2 but not the S1 antigen domain. This interesting observation prompted us to construct a vaccine vector delivering a modified stabilized version of the SARS-CoV-2 S antigen, with an inactivated S1/S2 cleavage site, called MVA-SARS-2-ST (for stabilized S antigen, MVA-ST) to compare with the original MVA-S in preclinical studies.

Here, we show that MVA-ST produces a full-length SARS-CoV-2 S protein that is not processed into S1 and S2 protein subunits, but anchored to the membrane of MVA-ST–infected cells. We found enhanced levels of cell-surface S1 antigen upon infection with MVA-ST compared with MVA-S. Moreover, when comparatively tested as a vaccine in animal models, MVA-ST not only elicited substantially higher levels of S1-binding and SARS-CoV-2–neutralizing antibodies, but also robustly protected vaccinated mice and hamsters against SARS-CoV‑2 respiratory infection and lung pathology. Currently, MVA-ST is being investigated in a phase Ib clinical trial as an optimized MVA vector candidate vaccine against COVID-19.

## Results

### Antibody response against different S protein domains in human volunteers vaccinated with MVA-S.

The MVA-based candidate vaccine MVA-S, encoding an unmodified, full-length SARS-CoV-2 S protein, is being tested in a phase Ia clinical study. This involved a prime-boost intramuscular vaccination schedule comparing low dose (1 × 10^7^ infectious units [IU]) versus high dose (1 × 10^8^ IU). The full prime-boost vaccination regimen was administered to 30 participants at an interval of 28 days. We collected blood from these individuals at several time points, including before vaccination (day 0), after the first vaccination (day 28), and at 2 time points after the second vaccination (days 42 and 84).

**Figure 1:**
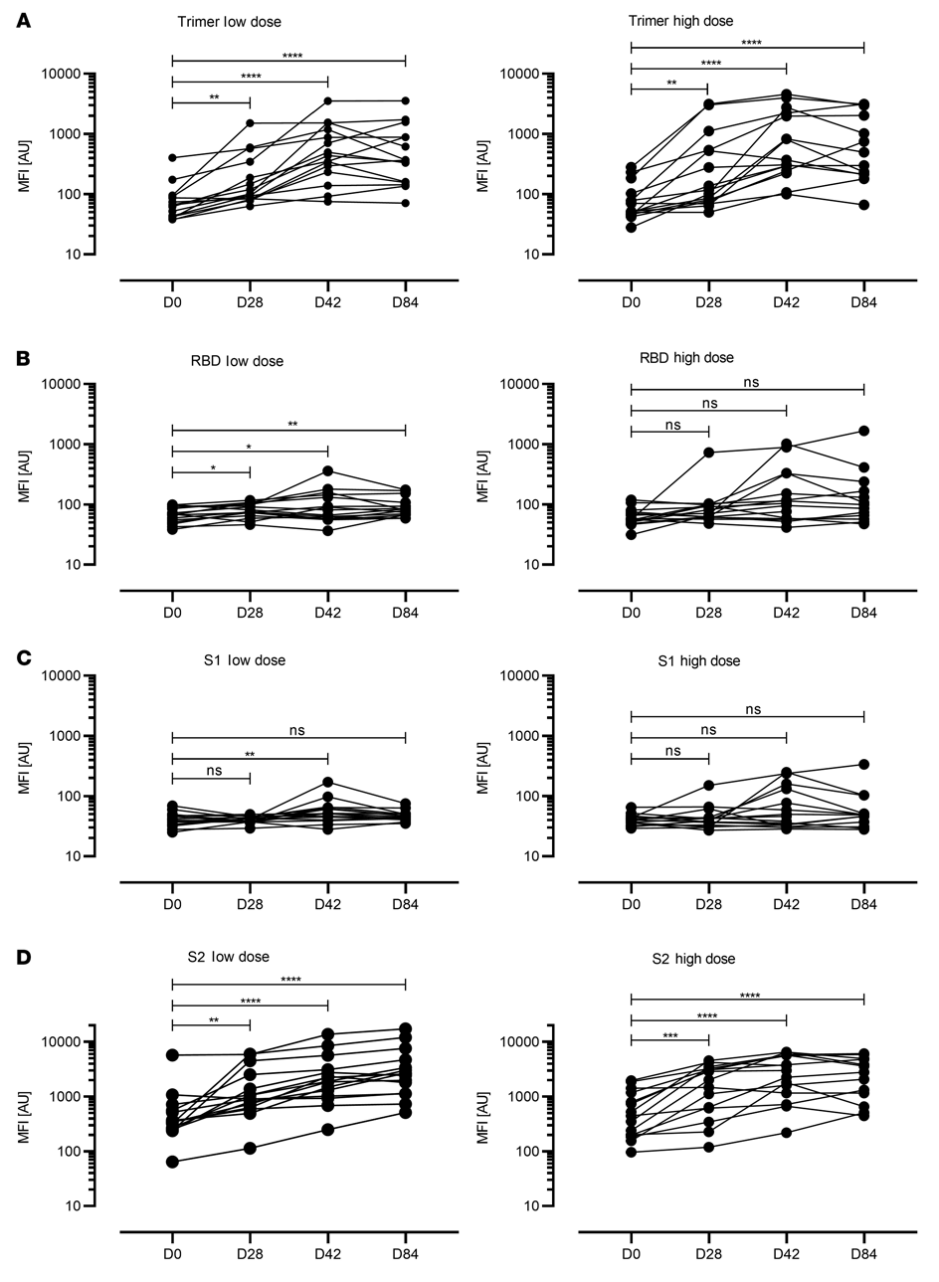
SARS-CoV-2–specific antibody responses in human volunteers vaccinated with MVA-S. Scatterplots represent data from individual participants. Humoral immunity against the SARS-CoV-2 spike protein domains were characterized using a multiplex bead array. Antibody reactivity was measured against (**A**) the full spike protein expressed as a trimeric antigen (S), (**B**) the receptor binding domain of the spike protein (RBD), (**C**) the S1 domain (S1), and (**D**) the S2 domain (S2). Antibody levels were quantified at baseline (BL), before vaccine boost (D28), 2 weeks after vaccine boost (D42), and 8 weeks after vaccine boost (D84) in the low- (left panels) and high-dose (right panels) groups. Seroconversion was estimated by comparison to a calibrator sample. Cutoff values: trimer = 1085 MFI, RBD = 640 MFI, S2 = 2 × BL MFI. *P < 0.05, **P < 0.01, ***P < 0.001, ****P < 0.0001 by 1-way ANOVA with Dunnett’s multiple comparisons test of log-transformed data.

To characterize the antigen binding capacities of the SARS-CoV-2–specific antibodies, we performed a high-throughput, automated bead-based multiplex assay called Multi-CoV-Ab (16, 17), where 4 different SARS-CoV-2–specific antigens (trimeric full-length S protein [S trimer], receptor-binding domain [RBD], and S1 and S2) are expressed and immobilized on LUMINEX MAGPLEX beads. Seroconversion was estimated by a comparison relative to a calibrator sample. To examine MVA-S–induced seroconversion, we used the trimer antigen assay (Figure 1A). All individuals vaccinated with the low dose mounted low levels of trimer-binding antibodies that peaked on day 42, with a mean titer expressed as a median fluorescence intensity (MFI) of 787.7. Thirty-three percent (*n* = 5/15) of the individuals reached antibody titers relevant for seroconversion. In the high-dose vaccination group, we detected marginally increased trimer-specific antibody responses with a mean titer of 1274 MFI peaking 2 weeks after the second vaccine dose, and 33.3% (*n* = 5/15) of the individuals seroconverted (Figure 1A).

When evaluating serum reactivity against the RBD of the SARS-CoV-2 S protein we found markedly lower quantities of antigen-binding antibodies (Figure 1B). Only 26.7% (*n* = 4/15) of the vaccinees receiving the high-dose immunization produced an anti-RBD response (with a peak mean titer of 232.9 MFI on day 42), and of these, only 2 individuals reached elevated RBD-specific antibody levels compared with the calibrator. In the sera from low-dose vaccinees, we did not detect any RBD-specific antibodies.

Analyzing the IgG response directed against the S1 and S2 subdomains of the SARS-CoV-2 spike protein (Figure 1, C and D) revealed marginal levels of S1-specific antibodies in a few individuals only, irrespective of the dosage used for vaccination (Figure 1C). In contrast, we measured substantial quantities of S2-binding antibodies in sera from all vaccinees, irrespective of the dosage used for vaccination (Figure 1D). Again, seroconversion was estimated by comparison relative to a calibrator sample. To avoid any false positive results due to extensive background fluorescence associated with the S2 subdomain, we defined the cutoff values as 2 × (day 0 MFI). The S2-specific antibody response peaked on day 84 in the low-dose group, with a mean titer of 4206.5 MFI. In the high-dose group, half of the vaccinees exhibited a peak on day 42 (mean titer of 3271.2 MFI), whereas the rest developed steadily increasing levels of SARS-CoV-2 S2–binding antibodies until day 84 (mean titer of 2928.9 MFI). Altogether, these results indicated that vaccination with the candidate vaccine MVA-S expressing a full-length unmodified S protein predominantly induces an S2-specific antibody response in humans.

### Generation and characterization of the modified candidate vaccine MVA-ST.

To investigate the possible impact of fusogenic activity and proteolytic cleavage of the native full-length S protein delivered by MVA-S, we generated a matching MVA vector vaccine producing a modified version of the SARS-CoV-2 S antigen, MVA-ST. To obtain an S antigen stabilized in a prefusion conformation we introduced 5 amino acid (aa) exchanges within the 1273-aa S polypeptide, inactivating the S1/S2 furin cleavage site and creating 2 new proline residues (K986P, V987P) between the first heptad repeat (HR1) and the central helix of the S2 protein (Supplemental Figure 1, A–C; supplemental material available online with this article; https://doi.org/10.1172/JCI159895DS1). The recombinant MVA-ST was clonally isolated in plaque purifications in DF-1 cell cultures and PCR analyses of the viral genome confirmed the genetic integrity and genetic stability of the vector virus (Supplemental Figure 1, D–G). The suitability of MVA-ST for production at industrial scale under conditions of biosafety level 1 was indicated by data from growth testing in DF-1 producer cells and in cell lines of human origin (Supplemental Figure 2).

**Figure 2:**
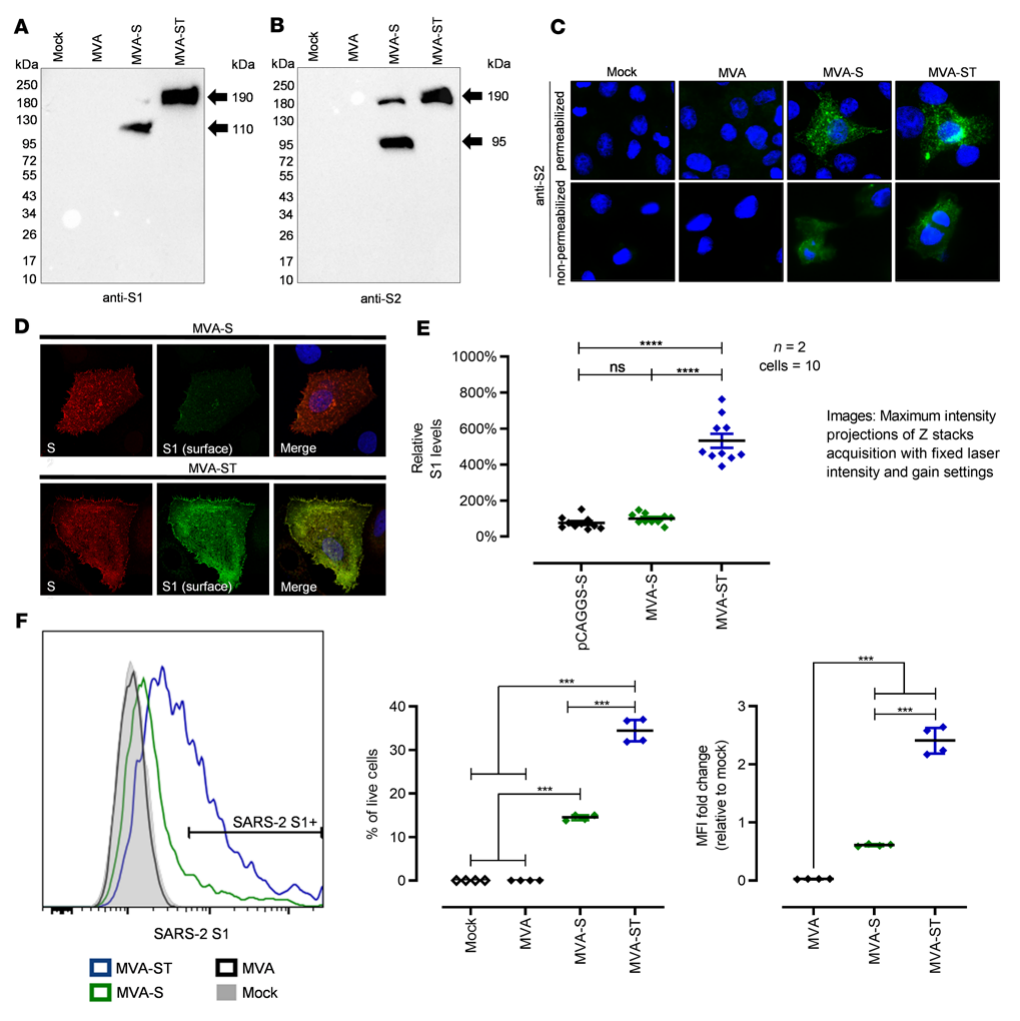
Synthesis and processing of spike glycoprotein (S) in MVA-S– and MVA-ST–infected cells. (**A** and **B**) Western blot analysis of S in lysates of MVA-S– and MVA-ST–infected cells. Noninfected (mock) or MVA-infected cells served as controls. DF-1 and Vero cells were infected with an MOI of 10 and collected 24 hours after infection. Polypeptides were resolved by SDS-PAGE and analyzed with a monoclonal antibody against (**A**) SARS-CoV-2 S1 or (**B**) SARS-CoV-2 S2. (**C**) Immunofluorescent staining of S in MVA-, MVA-S–, and MVA-ST–infected Vero cells (MOI = 0.5). Cells were permeabilized or nonpermeabilized and probed with mouse monoclonal antibodies against SARS-CoV-2 S protein (S2 domain, green). Cell nuclei were counterstained with DAPI (blue). (**D** and **E**) Immunofluorescent single-cell staining of surface S levels. Huh-7 cells were infected with MVA-S, MVA-ST, or transfected with plasmids encoding unmodified S (pCAGGS-S). (**D**) At 18 hours after infection, cell-surface S was labeled with anti-S1 monoclonal antibody and total S was labeled with anti-S2 antibody after fixation and permeabilization. Nuclei were counterstained with DAPI. Original magnification, ×100 (**C**) and ×630 (**D**). (**E**) For quantification, fluorescence intensity of surface S was measured and set in relation to that of total S. In total, 10 cells from 2 independent experiments were analyzed for each setup. (**F**) Flow cytometric analysis of surface S1 expression by MVA-S– or MVA-ST–infected A549 cells. Graphs show the percentage of S1^+^ cells (*n* = 4) and the fold change in S1 median fluorescence intensity (MFI) relative to the mock control (*n* = 4). ***P < 0.001, ****P < 0.0001 by 1-way ANOVA with Tukey’s multiple comparisons test.

Synthesis of the stabilized ST antigen in MVA-ST–infected cell cultures was demonstrated by Western blot analysis, which confirmed the absence of proteolytic cleavage. A single protein band with a molecular mass of approximately 190 kDa was detected in cells infected with MVA-ST using either S1- or S2-specific monoclonal antibodies (Figure 2, A and B). In contrast, lysates from cells infected with the original recombinant MVA-S contained additional protein bands that migrated at molecular masses corresponding to the sizes of the S1 and S2 cleavage products.

Next, we used immunofluorescent staining with S2-specific primary antibodies to assess cell surface expression and trafficking of the different S proteins in Vero cells infected with MVA-ST compared to cells infected with MVA-S (Figure 2C). Similar to our findings with MVA-S, we observed a reticular pattern with juxtanuclear accumulation of the stabilized S protein in permeabilized and MVA-ST–infected cells. Immunostaining without cell permeabilization specifically revealed abundant S2 protein on the cell surface of either MVA-S– or MVA-ST–infected cells.

To comparatively analyze and quantify predicted cellular localization of the S1 and S2 subunits by confocal microscopy, we infected Huh-7 cells with either MVA-S or MVA-ST (Figure 2, D and E). Infected cells were fixed 18 hours after infection and S located at the cell surface was labeled prior to fixation using an anti-S1 human-derived monoclonal antibody (18). Subsequently, cells were fixed, permeabilized, and total S was labeled using an anti-S2 antibody from mouse and secondary Alexa Fluor 488– and 594–conjugated antibodies (18). As anticipated, we saw a similar staining pattern for both recombinant viruses using S2-specific antibodies, indicating comparable amounts of S2 protein on the cell surface of both MVA-S– and MVA-ST–infected cells.

In MVA-ST–infected cells, the S1-specific immunostaining also revealed ample amounts of S1 protein on the cell surface. Surprisingly, and in contrast, we observed significantly lower levels of S1-specific cell surface staining in MVA-S–infected cells (Figure 2, D and E). Likewise, analyzing infected cells for S1 cell surface expression using immunostaining and FACS analysis detected significantly lower levels of S1 cell surface expression in cells infected with MVA-S (14.4%), in contrast to S1-specific staining in 34.5% of viable human A549 cells infected with MVA-ST. This was also confirmed when analyzing the fold change in S1 MFI relative to mock infection in the live cell compartment (0.61-fold change for MVA-S, 2.40-fold change for MVA-ST; Figure 2F).

### MVA-ST–induced S-specific immune responses in BALB/c mice.

To comparatively assess vaccine safety and immunogenicity, we vaccinated BALB/c mice intramuscularly with 1 × 10^8^ PFU of MVA-S or MVA-ST using a 21-day interval prime-boost schedule (Supplemental Figure 3).

**Figure 3:**
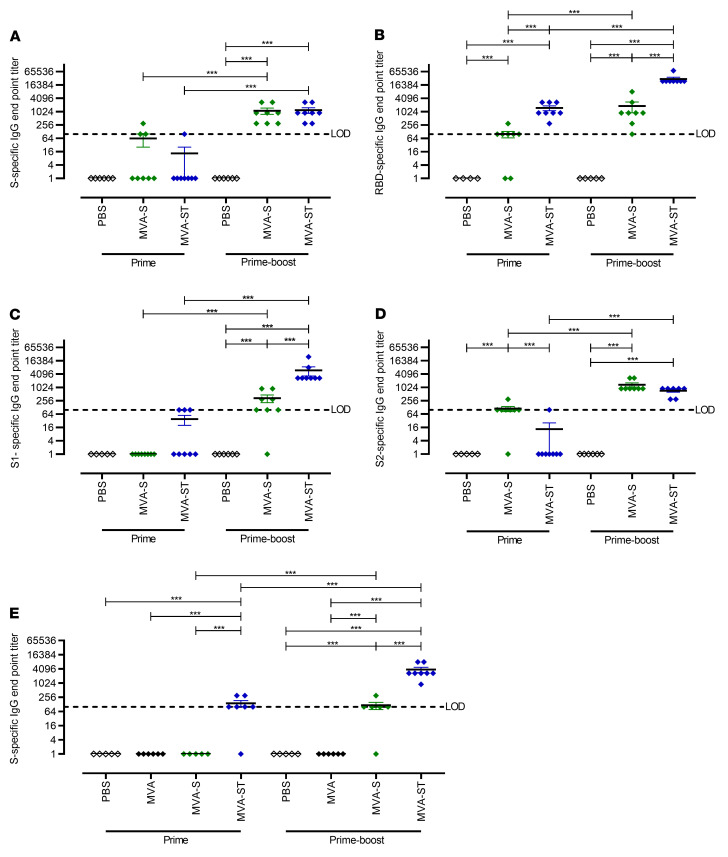
Antigen-specific humoral immunity induced by MVA-S or MVA-ST. BALB/c mice were i.m. vaccinated in a prime-boost regime (21-day interval) with 1 × 10^8^ PFU of MVA-S, MVA-ST, or PBS as controls. Sera were collected 18 days after the first immunization (prime *n* = 7–8) and 14 days after the second immunization (prime-boost *n* = 6–8). Sera were analyzed for SARS-CoV-2 S–binding antibodies in different ELISAs targeting the SARS-CoV-2 BavPat1 strain with (**A**) S-specific, (**B**) RBD-specific, (**C**) S1-specific, (**D**) S2-specific IgG antibodies, or targeting the Beta SARS-CoV-2 S (B1.351 variant) with (**E**) S-specific IgG antibodies. ***P < 0.001 by 1-way ANOVA with Tukey’s multiple comparisons test of log-transformed data. LOD, limit of detection.

The induction of S-binding antibodies was analyzed by ELISA using different SARS-CoV-2 S polypeptides as target antigens (full-length S, RBD, S1, or S2) (Figure 3, A–D). Initially, we confirmed seroconversion by ELISA using wells coated with purified trimeric S protein. Seroconversion was detected in 100% of vaccinated mice after prime-boost vaccination, with a mean titer of 1:1125 for MVA-S and 1:1200 for MVA-ST (Figure 3A). All MVA-ST–immunized mice already mounted antibodies binding to RBD on day 18, with a mean titer of 1:1500 (Figure 3B). Only 16.7% (*n* = 1/6) of MVA-S–vaccinated animals produced measurable amounts of RBD-specific antibodies, with a titer of 1:300. Boost vaccination on day 21 resulted in lower levels of RBD-specific antibodies following MVA-S vaccination (mean titer of 1:1850) than the significantly increased levels induced by MVA-ST vaccination (mean titer of 1:30,375).

In S1 ELISAs, no or only low-level responses were detected in sera of vaccinated mice after prime immunization (Figure 3C). We found that 37.5% (*n* = 3/8) of mice vaccinated with MVA-ST mounted S1-binding antibodies with a mean titer of 1:100. However, substantial levels of S1-binding antibodies developed after the boost vaccination with MVA-ST, with a mean titer of 1:6075; in contrast, mice that received MVA-S developed a significantly lower titer of 1:337. Marginal levels of antibodies binding to S2 protein were measured after a single vaccination with MVA-S or MVA-ST (Figure 3D). Boost vaccination significantly increased the amounts of S2-binding antibodies for both candidate vaccines, with a mean titer of 1:728 for MVA-ST–vaccinated and 1:1350 for MVA-S–vaccinated animals.

In addition, we analyzed antibody binding capacity against the Beta variant of SARS-CoV-2 using ELISA plates coated with synthetic Beta SARS-CoV-2 S protein (Figure 3E). A single MVA-S vaccination did not result in obvious levels of binding antibodies, whereas mice vaccinated with MVA-ST mounted detectable levels of binding antibodies, with a mean titer of 1:143. After boost vaccination, MVA-S–vaccinated mice did show activation of antibodies specific for the Beta variant S protein, with a mean titer of 1:116. However, MVA-ST booster immunization significantly increased these antibody levels, with a mean titer of 1:3825.

**Figure 4:**
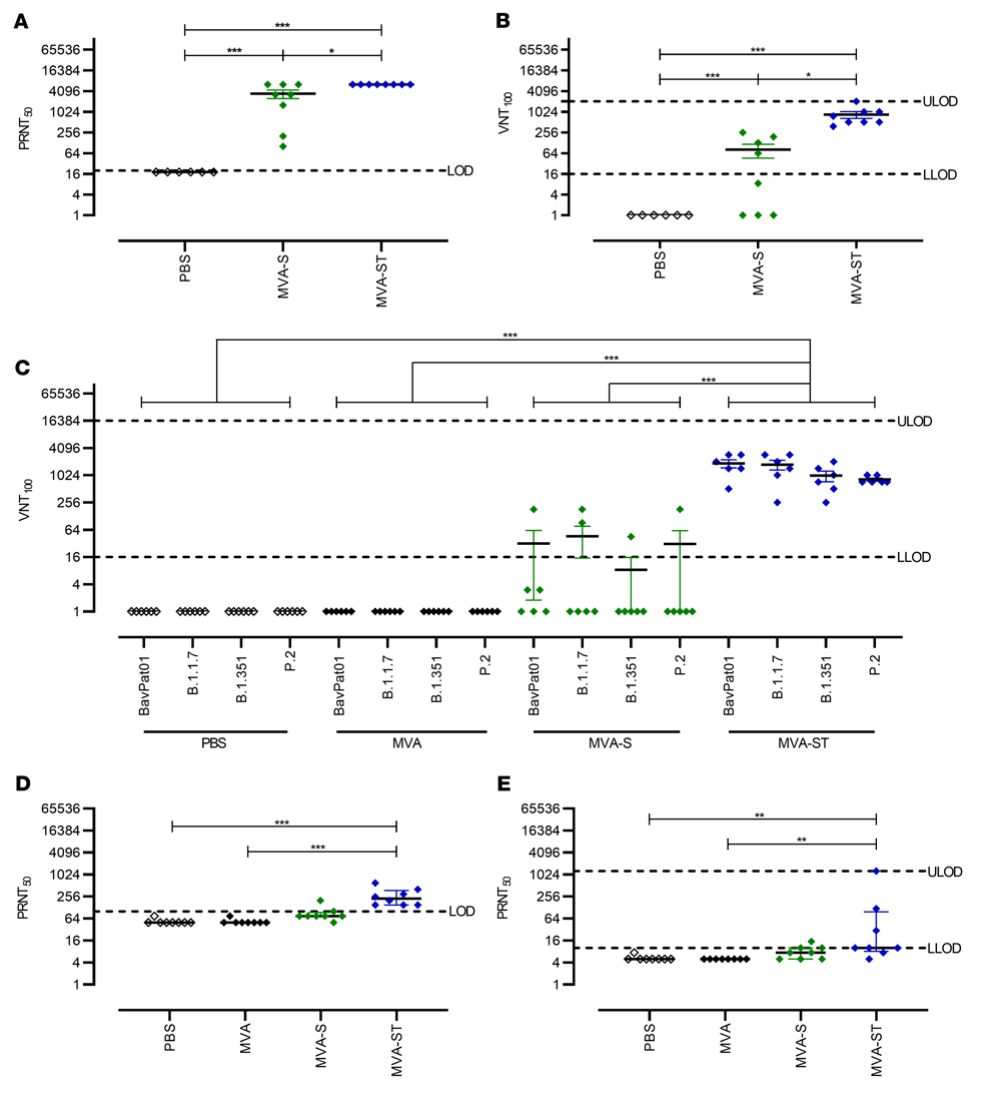
Virus-neutralizing antibody responses to SARS-CoV-2 BavPat1, Alpha, Beta, Zeta, Delta, and Omicron variants in vaccinated BALB/c mice. SARS-CoV-2 neutralization titers measured by the plaque reduction assay (PRNT_50_) and virus neutralization test (VNT_100_) from BALB/c mice vaccinated with PBS, MVA, MVA-S, or MVA-ST. (**A**) PRNT_50_ and (**B**) VNT_100_ assays using SARS-CoV-2 BavPat1. (**C**) VNT_100_ against SARS-CoV-2 BavPat1, Alpha, Beta, and Zeta variants. PRNT_50_ assay using SARS-CoV-2 (**D**) Delta and (**E**) Omicron variants. *P < 0.05, **P < 0.01, ***P < 0.001 by 1-way ANOVA with Tukey’s multiple comparisons test of log-transformed data (**A**–**C**) and Kruskal-Wallis test with Dunn’s multiple comparisons test (**D** and **E**). LOD, limit of detection; ULOD and LLOD, upper and lower LOD.

To evaluate neutralizing antibodies, we performed the 50% plaque reduction neutralization test (PRNT_50_) as well as the virus neutralization titer (VNT_100_) assay (Figure 4). Immunization with MVA-S induced low levels of neutralizing antibodies against the SARS-CoV-2 isolate Germany/BavPat1/2020 (henceforth called SARS-CoV-2 BavPat1), reaching a mean titer of 1:3437 in the more sensitive PRNT_50_ and a mean titer of 1:81 in the more demanding VNT_100_ assay (Figure 4, A and B). In comparison, MVA-ST prime-boost vaccination resulted in significantly better SARS-CoV-2 BavPat1 neutralization, with mean titers of 1:6400 in PRNT_50_ and 1:848 in the VNT_100_ assay (Figure 4, A and B).

Our candidate vaccines are based on the S protein sequence of the SARS-CoV-2 isolate Wuhan HU-1 from 2020 (15). Thus, we used the mouse sera generated above to evaluate the capacity of the antibody responses to neutralize infections with SARS-CoV-2 variants Alpha (B.1.1.7), Beta (B.1.351), and Zeta (P.2) using the VNT_100_ assay (Figure 4C). Similar to previous findings using this assay, MVA-S vaccination resulted in low levels of detectable neutralizing antibodies against the original SARS-CoV-2 BavPat1 (geometric mean titer 31). In accordance with these results, only a few mice mounted neutralizing responses against the SARS-CoV-2 variants Alpha (2/6, mean titer of 1:46), Beta (1/6, mean titer of 1:8), and Zeta (1/6, mean titer 1:31). In sharp contrast, MVA-ST vaccination elicited robust levels of circulating antibodies that neutralized the original SARS-CoV-2 BavPat1 (6/6, mean titer of 1:1874) and the variant viruses Alpha (6/6, mean titer of 1:1761), Beta (6/6, mean titer of 1:1002), and Zeta (6/6, mean titer of 1:824).

To characterize the neutralizing capacities against the more recent SARS-CoV-2 variants Delta (B.1.617.2) and the highly contagious Omicron (B.1.1.529), we again performed prime-boost vaccination in BALB/c mice as above (Figure 4, D and E). Control mice that had been either mock or nonrecombinant MVA vaccinated did not mount any neutralizing antibodies against Delta or Omicron. MVA-S–vaccinated mice mounted low levels of Delta-neutralizing antibodies, with a mean titer of 1:90. In contrast, MVA-ST vaccination resulted in robust activation of Delta-neutralizing antibodies, with a mean titer of 1:275. When analyzing neutralization against Omicron, MVA-S–vaccinated mice showed low titers resulting in a mean titer of 1:8, compared with MVA-ST–vaccinated mice, with a mean of 1:184. To ensure comparability with the BALB/c vaccination experiments above, PRNT_50_ against the BavPat1 isolate was performed (Supplemental Figure 4). Altogether, these results indicate that immunization with MVA-ST induces a superior anti–SARS-CoV-2-S humoral response resulting in the generation of cross-neutralizing anti–SARS-CoV-2 S antibodies against all the variants tested so far: Alpha, Beta, Zeta, Delta, and Omicron.

**Figure 5:**
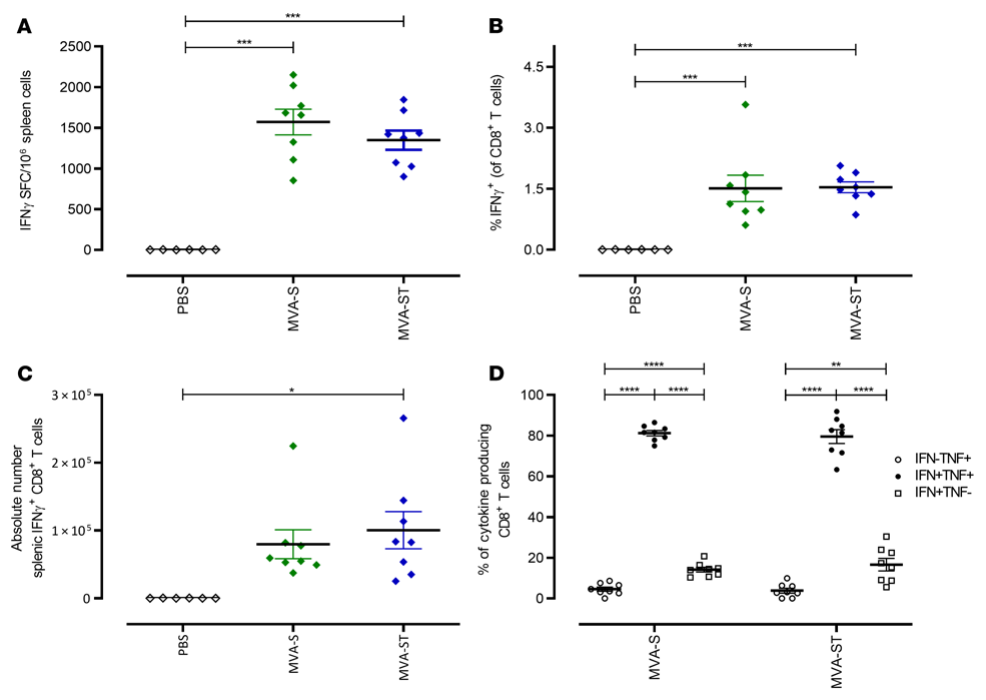
Activation of S-specific CD8^+^ T cells after prime-boost immunization with MVA-S or MVA-ST. Groups of BALB/c mice (*n* = 4–8) were immunized i.m. twice with 1 × 10^8^ PFU MVA-S, MVA-ST, or PBS as negative controls. (**A**–**D**) Splenocytes were collected and prepared on day 14 after boost immunization and stimulated with the H2-Kd–restricted peptide S_268–276_ (S1; GYLQPRTFL) and tested using ELISPOT assays and ICS FACS analyses. (**A**) IFN-γ^+^ SFC measured by ELISPOT assays. (**B** and **C**) IFN-γ–producing CD8^+^ T cells measured by FACS analysis. (**D**) IFN-γ–and TNF-α–producing CD8^+^ T cells measured by FACS analysis. *P < 0.05, **P < 0.01, ***P < 0.001, ****P < 0.0001 by 1-way ANOVA with Tukey’s multiple comparisons test.

To characterize the activation of SARS-CoV-2–specific cellular immunity following prime-boost vaccination in BALB/c mice, we monitored S1 epitope–specific CD8^+^ T cells using IFN-γ ELISPOT assays and FACS analysis (Figure 5). Boost vaccinations with MVA-S activated substantial numbers of S_268–276_ epitope–specific CD8^+^ T cells, with a mean number of 1571 IFN-γ^+^ spot-forming cells (SFC) in 1 × 10^6^ splenocytes (Figure 5A). Comparable results were obtained for boost vaccinations with MVA-ST (mean of 1349 IFN-γ^+^ SFC; Figure 5A). In agreement with these data, FACS analysis of T cells stimulated in vitro with peptide S_268–276_ and stained for intracellular IFN-γ showed robust frequencies of IFN-γ^+^CD8^+^ T cells in splenocytes from mice immunized with MVA-S (mean of 1.51%) or MVA-ST (mean of 1.53%) compared with mock-vaccinated control mice (mean of 0.01%) (Figure 5, B and C). Substantial numbers of the activated IFN-γ^+^CD8^+^ T cells also coexpressed TNF-α (81% for MVA-S and 79% for MVA-ST; Figure 5D). Of note, mice immunized with MVA-S or MVA-ST mounted similar levels of SARS-CoV-2 S–specific CD8^+^ T cells and MVA-specific CD8^+^ T cells (Supplemental Figure 5).

### Protective capacity of MVA-S and MVA-ST upon SARS-CoV-2 respiratory challenge in Syrian hamsters.

**Figure 6:**
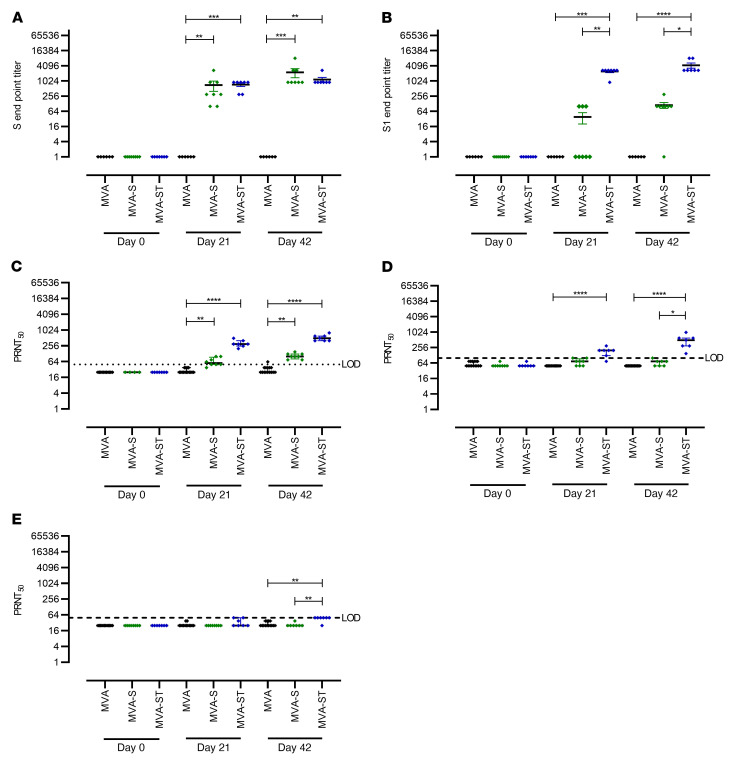
Antigen-specific humoral immunity induced in MVA-S– or MVA-ST–vaccinated hamsters. Syrian hamsters (*n* = 7–8) were i.m. vaccinated twice (21-day interval) with 1 × 10^8^ PFU of MVA-S (*n* = 8), MVA-ST (*n* = 7), or MVA (*n* = 15) as controls. Sera were collected on days 0, 21, and 42 and analyzed for SARS-CoV-2 S–binding antibodies in ELISAs targeting SARS-CoV-2 BavPat1 with (**A**) S-specific and (**B**) S1-specific IgG antibodies. SARS-CoV-2–neutralizing antibodies against SARS-CoV-2 (**C**) BavPat1, (**D**) Delta, and (**E**) Omicron variants were analyzed by PRNT_50_. *P < 0.05, **P < 0.01, ***P < 0.001, ****P < 0.0001 by Kruskal-Wallis test with Dunn’s multiple comparisons test. LOD, limit of detection.

**Figure 7:**
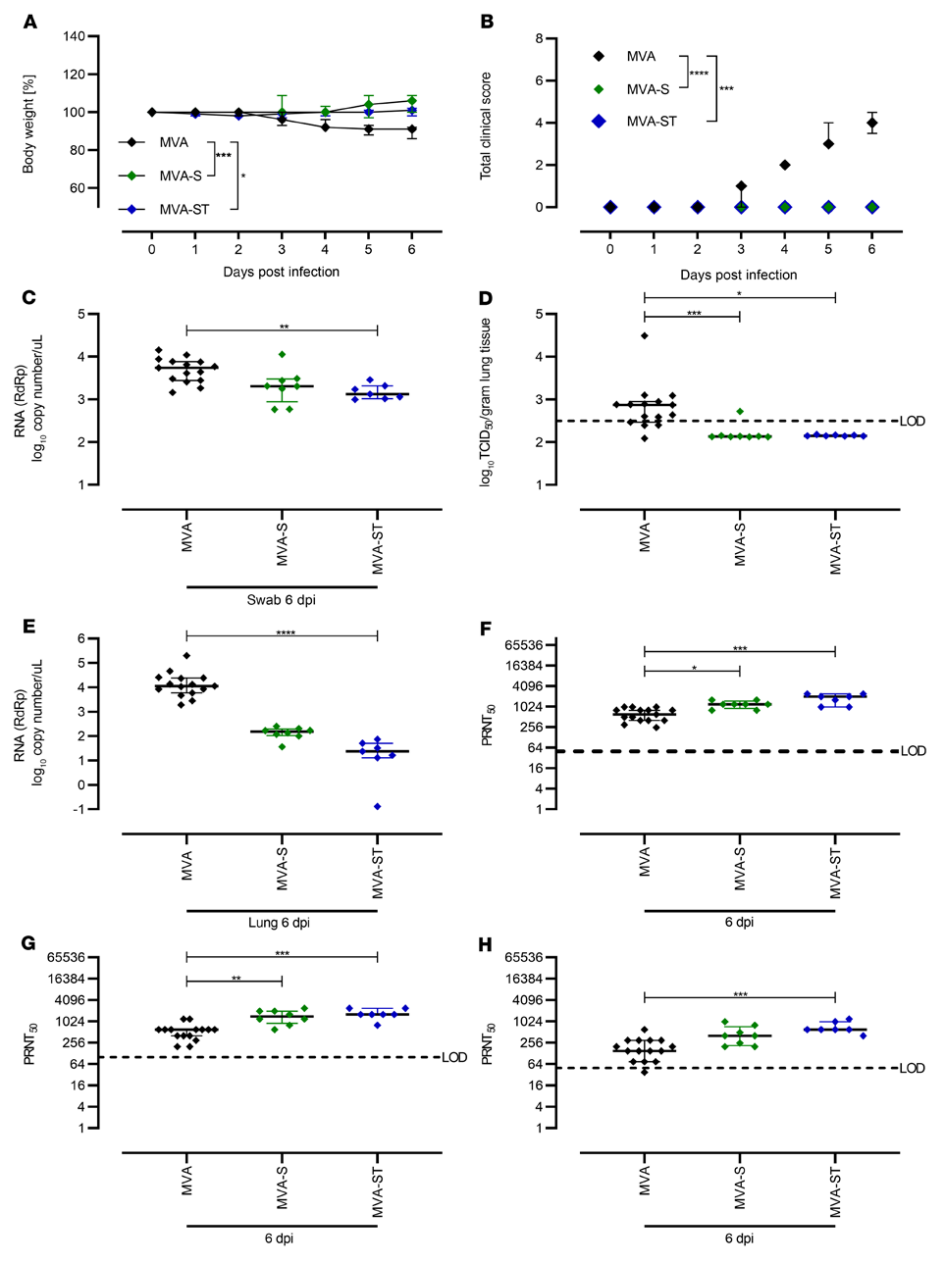
Protective capacity of MVA-S or MVA-ST immunization against SARS-CoV-2 BavPat1 infection in Syrian hamsters. Syrian hamsters vaccinated with MVA (*n* = 15) control, MVA-S (*n* = 8), or MVA-ST (*n* = 7) were i.n. challenged with 1 × 10^4^ TCID_50_ SARS-CoV-2 BavPat1. (**A**) Body weight was monitored daily and (**B**) spontaneous behavior and general condition were evaluated by clinical scores. (**C**) Oropharyngeal swabs on day 6 after challenge infection were analyzed for SARS-CoV-2 gRNA copies. (**D** and **E**) Lungs were harvested and analyzed for (**D**) infectious SARS-CoV-2 by TCID_50_/gram lung tissue, and (**E**) SARS-CoV-2 gRNA copies. Sera were prepared on day 6 after challenge and analyzed for SARS-CoV-2 (**F**) BavPat1, (**G**) Delta, and (**H**) Omicron variant–neutralizing antibodies by PRNT_50_. *P < 0.05, **P < 0.01, ***P < 0.001, ****P < 0.0001 by Kruskal-Wallis test with Dunn’s multiple comparisons test (**C**–**H**) of AUC (**A** and **B**). LOD, limit of detection.

To further investigate the impact of prime-boost immunization against SARS-CoV-2–induced disease, we used Syrian hamsters as a well-established preclinical model for efficacy testing (Figures 6 and 7). Two cohorts of hamsters were vaccinated within a 21-day interval twice intramuscularly with 1 × 10^8^ PFU candidate vaccine in each case, comparing MVA and MVA-S and then MVA and MVA-ST. Safety and immunogenicity were analyzed as established before (Supplemental Figure 6). SARS-CoV-2–binding antibodies were analyzed by different ELISAs specific for trimeric S protein or S1 subunit antigen. Immunizations with nonrecombinant vector elicited no detectable S-specific antibodies in control hamsters (MVA; Figure 6A). However, antibodies specific for trimeric S proteins could be detected in all hamsters vaccinated with MVA-S (mean titer 1:700) or MVA-ST (mean titer 1:728) already after single vaccination. Boost vaccinations further increased the levels, resulting in comparable titers of 1:2250 for MVA-S and 1:1157 for MVA-ST vaccination.

Underlining the mouse model results, we observed a different pattern for vaccine-induced S1-binding antibodies. Only 37.5% (*n* = 3/8) of MVA-S–vaccinated hamsters mounted S1-binding antibodies after the first immunization (mean titer of 1:38), while boost vaccinations elicited low-level seroconversion in 87.5% (*n* = 7/8) of MVA-S–vaccinated animals (mean titer of 1:112). In sharp contrast, prime MVA-ST vaccination induced high levels of S1-binding antibodies (100% seroconversion, mean titer of 1:2442), and boost vaccination on day 21 further increased these levels to a mean titer of 1:4242 (Figure 6B).

Similarly, after prime immunization we measured low levels of SARS-CoV-2 BavPat1–neutralizing antibodies in sera from 87.5% (*n* = 7/8) of MVA-S–vaccinated hamsters (mean titer of 1:65; Figure 6C), whereas all hamsters immunized with MVA-ST mounted readily detectable neutralizing antibodies (100% seroconversion), with an average titer of 1:321 PRNT_50_ at 3 weeks after priming (Figure 6C).

Compared with SARS-CoV-2 BavPat1, reduced neutralizing activity against Delta and Omicron were measured. MVA-S vaccination resulted in marginal antibody titers neutralizing Delta (mean titer of 1:71; Figure 6D). No detectable titers against Omicron were measured after prime MVA-S vaccination (Figure 6E). Hamsters that had been vaccinated with MVA-ST mounted a mean titer of 1:185.7 against Delta (Figure 6D) and a titer below the detection limit against Omicron (mean titer of 1:33.9; Figure 6E). After the boost vaccination, sera from all MVA-S–vaccinated hamsters (100% seroconversion) revealed low neutralizing activity, with minor titers of approximately 1:100 PRNT_50_ against SARS-CoV-2 BavPat1 (Figure 6C). One out of 7 animals had confirmed seroconversion against Delta, exhibiting a mean titer of 1:67 after boost vaccination (Figure 6D). In MVA-S–vaccinated animals, no seroconversion was detected against Omicron (Figure 6E). In contrast, in all sera from hamsters vaccinated with MVA-ST we detected increased amounts of SARS-CoV-2–neutralizing antibodies against SARS-CoV-2 BavPat1 after the boost immunization, with a mean titer of 1:529 PRNT_50_ (Figure 6C). For Delta, a mean titer of 1:500 was measured in these vaccinated animals (100% seroconversion; Figure 6D), whereas no obvious titers of Omicron-neutralizing antibodies were detected in MVA-ST–vaccinated animals (mean titer of 1:46; Figure 6E).

Four weeks after the boost immunization, the animals were intranasally infected with 1 × 10^4^ 50% tissue culture infectious dose (TCID_50_) SARS-CoV-2 BavPat1 (Figure 7). Starting on day 3, MVA-vaccinated control hamsters demonstrated reduced body weights, and at 6 days after infection all animals had lost approximately 10% of their initial body weight. No body weight loss could be detected for hamsters immunized with MVA-S or MVA-ST (Figure 7A). Control animals also showed characteristic clinical symptoms associated with SARS-CoV-2 respiratory tract infection, including labored breathing, reduced activity, and scruffy fur. No MVA-S– or MVA-ST–vaccinated animals showed any signs of clinical disease (Figure 7B).

To evaluate viral loads and pathological changes in lung tissues, we euthanized all animals at 6 days after infection. Blood and swab samples were taken at necropsy, and lungs were harvested for further analysis. Substantial amounts of viral RNA were detected in oropharyngeal swabs of control animals (mean of 7.7 × 10^3^ RNA copy numbers/μL; Figure 7C). Swab samples from hamsters vaccinated with MVA-S contained marginally reduced levels of viral RNA (on average 3 × 10^3^ RNA copy numbers/μL), whereas swabs from animals vaccinated with MVA-ST contained significantly reduced levels of SARS-CoV-2 RNA (mean of 1.6 × 10^3^ RNA copy numbers/μL; Figure 7C).

Correspondingly, lung tissues from control hamsters harbored infectious SARS-CoV-2 (mean of 2.9 × 10^3^ TCID_50_/gram lung tissue; Figure 7D), whereas no infectious SARS-CoV-2 was detected in the lungs of vaccinated hamsters (with the exception of tissue from 1 MVA-S–vaccinated animal containing 5.6 × 10^2^ TCID_50_/gram lung tissue). These data were confirmed by real-time RT-PCR analysis of viral RNA loads. In lung tissues from both MVA-S– and MVA-ST–immunized animals, we found lower levels of SARS-CoV-2 RNA compared with control hamsters (<3 × 10^1^ genome equivalents/ng total RNA; Figure 7E).

Only after SARS-CoV-2 BavPat1 infection did we detect SARS-CoV-2–binding antibodies in control (MVA) hamsters, with a mean titer of 1:16,883 for S-specific antibodies and 1:5600 for S1-binding antibodies (Supplemental Figure 7, A and B). Thus, although all the control hamsters became moribund, we observed detectable titers of SARS-CoV-2 BavPat1–neutralizing antibodies that averaged to 1:632 PRNT_50_ after challenge infection (Figure 7F). Against Delta, an average mean titer of 1:1013 was measured in control MVA-vaccinated hamsters (Figure 7G). Lower titers reaching a mean of 1:204 were present against Omicron detected by PRNT_50_ (Figure 7H). In line with data from viral load and clinical disease outcome, we detected markedly higher levels of SARS-CoV-2 S–specific antibodies in sera from immunized hamsters. After challenge, we measured substantial levels of S-binding antibodies, with a mean titer of 1:38,185 or 1:50,194 after MVA-S or MVA-ST immunization (Supplemental Figure 7B). S1-binding antibodies in MVA-S–vaccinated hamsters reached a mean titer of 1:23,528; MVA-ST–vaccinated hamsters had a higher mean titer of 1:72,900 (Supplemental Figure 7A). MVA-S vaccination resulted in SARS-CoV-2 BavPat1–neutralizing activities with an average PRNT_50_ titer of 1:1200, compared with the MVA-ST mean titer of 1:1771 (Figure 7F). In MVA-S–vaccinated hamsters, a mean titer of 1:1475 against Delta and 1:468 against Omicron were detected (Figure 7, G and H). After MVA-ST vaccination, hamsters mounted mean titers of 1:1714 against Delta and 1:714 against Omicron (Figure 7, G and H).

**Figure 8:**
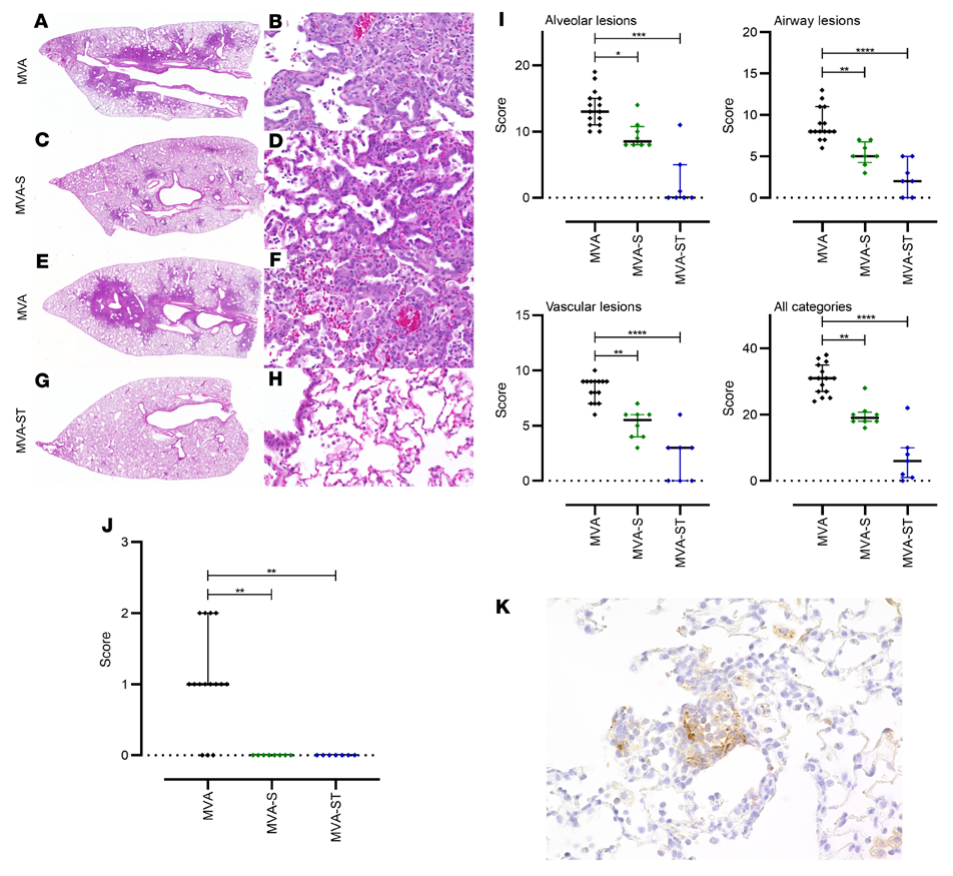
Histopathological lesions in the lungs of SARS-CoV-2 BavPat1–challenged hamsters vaccinated with MVA, MVA-S, or MVA-ST. (**A**, **C**, **E**, and **G**) Representative overview images of hematoxylin and eosin–stained lung sections and (**B**, **D**, **F**, and **H**) associated ×100 magnifications. (**A** and **E**) Images from MVA control–vaccinated animals show extensive areas of alveolar consolidation (arrowheads). Higher magnification (**B** and **F**) reveals markedly thickened alveolar septae, inflammatory infiltrates, and prominent pneumocyte type II hyperplasia with many atypical, large cells (arrowheads) and mitotic figures (arrow). (**C** and **D**) MVA-S–vaccinated animals show less lung pathology with multifocal, small foci of alveolar consolidation, which are qualitatively similar to the lesions in controls. (**G** and **H**) Most MVA-ST–vaccinated animals show no alveolar lesions. (**E**) Quantification of histopathological lesions. Vaccination with recombinant MVAs significantly reduces lung lesions compared with control groups. (**J** and **K**) Immunohistochemistry for SARS-CoV-2 nucleoprotein in the lungs of hamsters vaccinated with MVA (control), MVA-S, or MVA-ST, challenged with SARS-CoV-2 BavPat1. (**J**) Semiquantitative scoring of viral antigen amount. No viral antigen was detected in MVA-S– or MVA-ST–vaccinated animals. (**K**) SARS-CoV-2 antigen (brown signal) is predominantly found in pneumocytes lining alveoli (×100 magnification). Dotted lines mark the zero value. *P < 0.05, **P < 0.01, ***P < 0.001, ****P < 0.0001 by Kruskal-Wallis test with Dunn’s multiple comparisons test.

To evaluate lung pathology in vaccinated and infected animals, we performed histological analysis of hematoxylin and eosin–stained lung sections (Figure 8). Control hamsters (MVA) had large areas of lung consolidation. Alveolar lesions were characterized by the accumulation of neutrophils and mononuclear cells that expanded alveolar septae and filled alveolar lumina (Figure 8, A and E). Inflammation was associated with necrosis of alveolar epithelia, fibrin exudation, and a prominent pneumocyte type II hyperplasia. A mixed inflammatory infiltrate, epithelial degeneration, and hyperplasia were found in bronchi and bronchioli. In addition, animals showed marked vascular lesions, characterized by endothelial hypertrophy, endothelialitis, mural and perivascular infiltrates, loss of vascular wall integrity, and perivascular edema.

MVA-S–vaccinated hamsters also revealed areas of inflammation and consolidation, although the overall extent of alveolar, bronchial/bronchiolar, and vascular lesions was less than in control animals (Figure 8C). Lungs of MVA-ST–vaccinated hamsters showed negligible or markedly reduced lung pathology (Figure 8G). Almost all the animals in this group demonstrated only mild to moderate inflammatory lesions confined to the airways and some vessels, while alveolar lesions were absent or minimal, affecting less than 1% of the lung lobes. Only 1 animal showed higher lesion scores in the alveolar and vascular compartment, affecting below 25% of the entire lobe.

Semiquantitative scoring of alveolar, airway, and vascular lesions showed a significant reduction in all parameters in animals vaccinated with recombinant MVA vaccines compared with the control group (Figure 8I). Importantly, MVA-ST–vaccinated hamsters showed substantially lower inflammation scores than MVA-S–vaccinated animals. Using immunohistochemistry, SARS-CoV-2 nucleoprotein was detected in the lungs of all control hamsters, but in none of the MVA-S– or MVA-ST–immunized animals (Figure 8, J and K).

### MVA-S or MVA-ST vaccination provides protection from lethal SARS-CoV-2 disease outcomes in K18-hACE2 mice.

To evaluate immunogenicity and protective efficacy in a lethal animal model, we used K18-hACE2 mice. K18-hACE2 mice are highly susceptible to intranasal SARS-CoV-2 infection characterized by high viral loads in the lungs, severe interstitial pneumonia, and death by day 6 or 8 after inoculation. Mice were vaccinated with MVA, MVA-S, or MVA-ST using an intramuscular prime-boost schedule as above.

**Figure 9:**
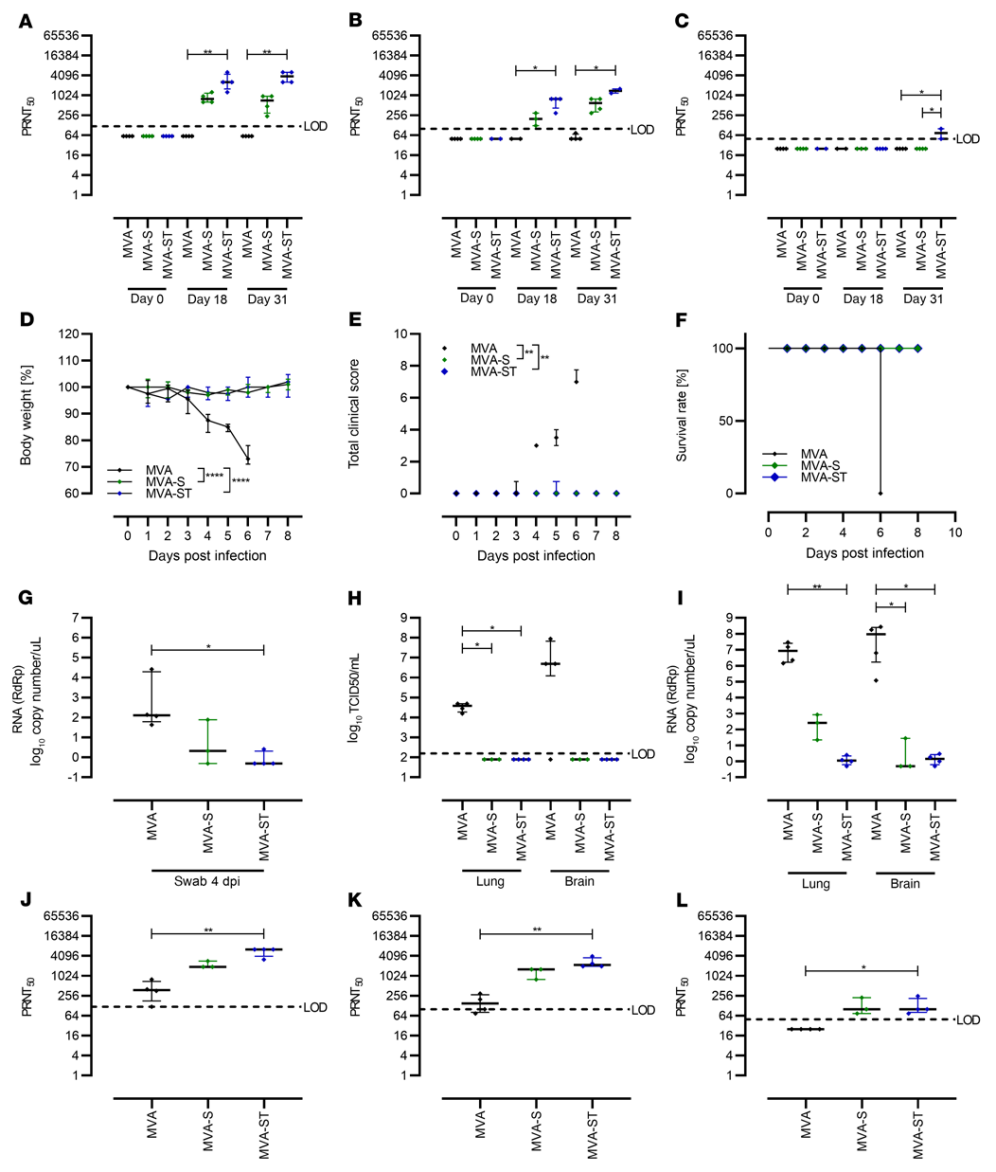
Protective capacity of MVA-S or MVA-ST immunization against SARS-CoV-2 in K18-hACE2 mice. K18-hACE2 mice were i.m. immunized twice with 1 × 10^8^ PFU MVA-S (*n* = 4), MVA-ST (*n* = 4), or MVA (*n* = 4) as a control in a 21-day interval. Sera were collected on days 0, 18, and 31 and analyzed for SARS-CoV-2–neutralizing antibodies against (**A**) BavPat1, (**B**) Delta, and (**C**) Omicron variants by PRNT_50_. After SARS-CoV-2 BavPat1 challenge infection, (**D**) body weight was monitored daily, (**E**) spontaneous behavior and general condition were evaluated in clinical scores, and (**F**) survival rate was determined retrospectively. (**G**) Oropharyngeal swabs from 4 days after infection were analyzed for SARS-CoV-2 gRNA copies. RdRp, RNA-dependent RNA polymerase. At the end of the experiment (day 6 for MVA-, day 8 for MVA-S/MVA-ST–vaccinated mice), lungs and brains were harvested and analyzed for (**H**) amounts of infectious SARS-CoV-2 by TCID_50_/mL and (**I**) viral RNA by qRT-PCR. Sera were analyzed for (**J**) BavPat1, (**K**) Delta, and (**L**) Omicron variant–neutralizing antibodies by PRNT_50_. *P < 0.05, **P < 0.01, ****P < 0.0001 by Kruskal-Wallis test with Dunn’s multiple comparisons test (**A**–**C** and **G**–**L**) of AUC (**E**) and 1-way ANOVA with Tukey’s multiple comparisons test of AUC (**D**). LOD, limit of detection.

As expected, we did not detect SARS-CoV-2 BavPat1–neutralizing antibodies in control mice vaccinated with MVA. Single vaccination with MVA-S or MVA-ST resulted in obvious titers of neutralizing antibodies against SARS-CoV-2 BavPat1, with a mean titer of 1:880 for MVA-S and 1:2880 for MVA-ST. Boost vaccination on day 21 further increased SARS-CoV-2 BavPat1–neutralizing antibodies to a mean titer of 1:660 or 1:3840 in MVA-S– or MVA-ST–vaccinated mice (Figure 9A). However, neutralizing activities against SARS-CoV-2 Delta and Omicron were lower compared with SARS-CoV-2 BavPat1 following MVA-S and MVA-ST vaccination (Figure 9, B and C).

Mice immunized with MVA-S mounted sufficient levels of Delta-neutralizing antibodies after prime or boost application (Figure 9B; mean of 1:208 or 1:575). MVA-ST vaccination resulted in a mean titer of 1:675 after prime and 1:1400 after boost (Figure 9B). For Omicron, no detectable titers of neutralizing antibodies were present in mice after single vaccination with either candidate vaccine. MVA-S boost vaccination again did not result in obvious titers of Omicron-neutralizing antibodies (Figure 9C). Marginal titers of Omicron-neutralizing antibodies were present in sera of mice after boost vaccination with MVA-ST (Figure 9C; mean titer of 1:75).

At 4 weeks after boost vaccinations, mice were intranasally challenged with a lethal dose of 3.6 × 10^4^ TCID_50_ SARS-CoV-2 BavPat1. Control mice significantly lost weight and showed clinical signs of disease starting on day 3, and all succumbed to infection by day 6, whereas MVA-S– and MVA-ST–vaccinated mice showed no weight loss or clinical disease (Figure 9, D–F). At 4 days after infection, substantial levels of viral RNA shedding were observed from the upper respiratory tract of control vaccinated mice (mean of 6.6 × 10^3^ genome equivalents/μL). In MVA-S–vaccinated mice, we found low but detectable levels of SARS-CoV-2 RNA shedding in oropharyngeal swabs (mean of 27 genome equivalents/μL). MVA-ST–vaccinated mice did not produce detectable viral RNA levels in oropharyngeal swabs (Figure 9G).

When monitoring viral loads in the lung and brain homogenates of mice at time of death (day 6 after infection [MVA-vaccinated mice] or 8 days after challenge [MVA-S/MVA-ST–vaccinated animals]), we failed to detect SARS-CoV-2 BavPat1 in the lungs and brains of MVA-S– or MVA-ST–vaccinated mice, but found large amounts of infectious virus in the organs from control MVA-vaccinated mice (Figure 9H). These data were confirmed by real-time RT-PCR analysis of viral RNA loads. In the control MVA-vaccinated mice, we detected substantial levels of viral RNA, with a mean of 1.19 × 10^7^ or 1.14 × 10^8^ genome equivalents/ng total RNA in lungs or brains. Both MVA-S– and MVA-ST–immunized animals exhibited lower levels of SARS-CoV-2 RNA than control mice in the lungs (a mean of 3.7 × 10^2^ genome equivalents/ng total RNA for MVA-S and 1.31 for MVA-ST; Figure 7I) and in the brains (a mean of 9.73 genome equivalents/ng total RNA for MVA-S and 1.58 for MVA-ST).

Neutralizing antibodies against ancestral SARS-CoV-2 BavPat1, SARS-CoV-2 Delta, and Omicron were analyzed at the end of the experiment. Marginal titers of BavPat1- and Delta-neutralizing antibodies were present in sera of control MVA-vaccinated mice after SARS-CoV-2 BavPat1 challenge infection (mean of 1:420 for BavPat1, mean of 1:168.75 for Delta; Figure 9, J and K). No titers of Omicron-neutralizing antibodies were found in MVA-vaccinated animals (Figure 9L). However, robust titers of neutralizing antibodies were present in sera of MVA-S– and MVA-ST–vaccinated mice after SARS-CoV-2 BavPat1 challenge infection (Figure 9, J–L). Against BavPat1, MVA-S vaccination resulted in a mean titer of 1:2240; MVA-ST vaccination resulted in an even higher mean titer of 1:5600.

Against Delta, MVA-S vaccination resulted in a mean titer of 1:1333. Confirming previous results, antibody levels in MVA-ST–vaccinated mice were markedly higher, with a mean titer of 1:2400. However, against Omicron, a lower mean titer of 1:133 was measured for both candidate vaccines (Figure 9L).

**Figure 10:**
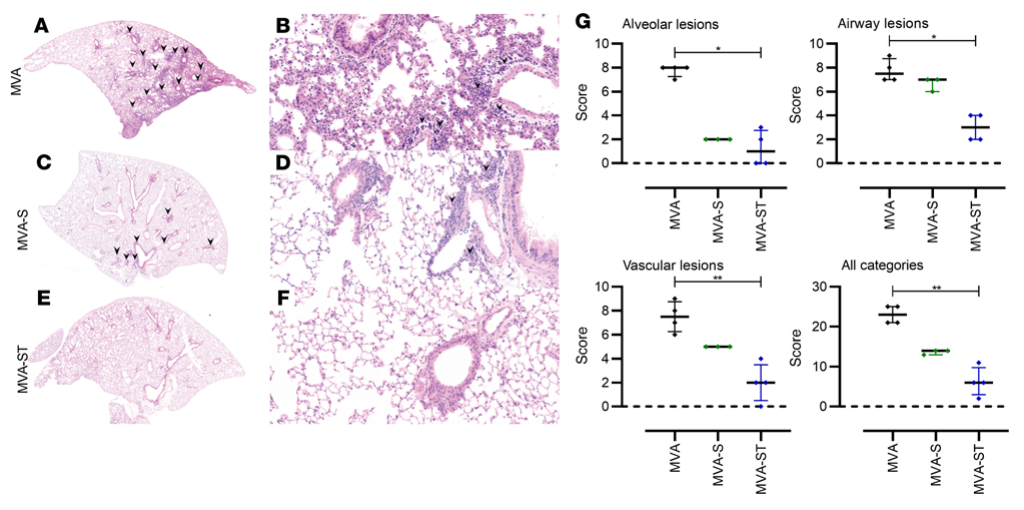
Histopathological lesions in the lungs of K18-hACE2 mice vaccinated with MVA, MVA-S, or MVA-ST, challenged with SARS-CoV-2 BavPat1. (**A**, **C**, and **E**) Representative overview images of hematoxylin and eosin–stained lung sections and (**B**, **D**, and **F**) associated ×100 magnifications. (**A**) MVA control–vaccinated animals show multifocal areas of immune cell infiltration (arrowheads). (**B**) Higher magnification reveals markedly thickened alveolar septae, inflammatory infiltrates, and a prominent perivascular immune cell infiltration (arrowheads) as well as multifocal perivascular edema. (**C** and **D**) MVA-S–vaccinated animals show less lung pathology with multifocal, small foci of thickened alveolar septae and mild to moderate perivascular infiltrates. (**E** and **F**) Most MVA-ST–vaccinated animals show no alveolar and fewer vascular lesions. (**G**) Quantification of histopathological lesions. Vaccination with recombinant MVAs reduces lung lesions compared with the control MVA group. Dotted lines mark the zero value. *P < 0.05, **P < 0.01 by Kruskal-Wallis test with Dunn’s multiple comparisons test.

Consistent with data from viral load in the lungs, control MVA-vaccinated animals showed pronounced lung pathology, which was associated with moderate to severe perivascular edema and inflammation with lymphocytes, macrophages, and small numbers of neutrophils surrounding small and intermediate vessels. Considerable inflammatory changes were also found in the alveolar and peribronchiolar compartments, characterized by moderate to marked interstitial and luminal immune cell infiltrates, with multifocal areas of completely obscured alveolar architecture. In animals vaccinated with MVA-S, despite the absence of severe and widespread inflammation in the alveolar compartment, substantial perivascular and peribronchiolar inflammation was also present in the lungs. Interestingly, MVA-ST–vaccinated mice showed only very mild signs of pulmonary lesions after SARS-CoV-2 BavPat1 challenge infection (Figure 10). Our data so far showed that robust protective vaccination by a prime-boost application of 1 × 10^8^ PFU MVA candidate vaccines is associated with substantial titers of neutralizing antibodies in K18-hACE2 mice.

## Discussion

Here, we report that vaccination with a prefusion, stabilized SARS-CoV-2 S protein (ST) expressed by recombinant MVA (MVA-ST) elicits a better humoral immune response and provides protection upon SARS-CoV-2 BavPat1 challenge infection compared with the original recombinant MVA vaccine delivering the nonmodified SARS-CoV-2 S antigen (MVA-S).

Although several approved vaccines against SARS-CoV-2 are currently available, COVID-19 vaccine development still remains an important goal due to unsolved questions such as longevity and duration of immunity, virus transmission after asymptomatic infection, and arising virus variants of concern (VOCs). Thus, the development of innovative vaccination modalities that also confer robust and more broadly effective protection are urgently required. In general, there are several strategies to further improve vaccine candidates. A promising approach includes the presentation of a selected antigen. This may be of importance when processable fusion proteins are used as immunogens in vaccine development, since their metastability or processing also affects the kinetics of immune responses.

Based on our positive experience using a nonmodified S protein for generating an MVA-based candidate vaccine against MERS-CoV (11), we initially decided to use the same strategy to develop a COVID-19 candidate vaccine. Our MVA-S candidate vaccine expressing the authentic 2019 Wuhan Hu-1 S protein was confirmed to be immunogenic and protective in preclinical evaluation when tested in a mouse model for COVID-19 (15). Comparable results have been reported from the Oxford-AstraZeneca ChAdOx1 nCoV-19 vaccine, which also expresses a nonstabilized S protein and was confirmed to be immunogenic and protective in different preclinical animal models (19–21). Of note, the Oxford-AstraZeneca ChAdOx1 nCoV-19 was approved as a COVID-19 vaccine for application in humans and more than 2 billion doses of the vaccine have already been administered (22, 23).

In this current study, we report on the evaluation of the COVID-19 candidate vector vaccine MVA-S in a phase Ia clinical trial in humans. Here, we again confirmed advantageous safety and tolerability (data not shown). However, preliminary results revealed a pattern of antibody responses in individuals vaccinated with high or low doses of MVA-S, indicating a low S1-specific antibody response irrespective of vaccine dosage, while these individuals mounted substantial titers of S2-binding antibodies. Overall, levels of S-specific antibodies were shown to be below the levels of a comparable study evaluating an MVA-based candidate vaccine against MERS-CoV (11).

A recent study indicated that the efficiency of furin-mediated cleavage in the S1/S2 polybasic cleavage site in SARS-CoV-2 is enhanced compared with MERS-CoV (24). From these preliminary in vitro results, we hypothesize that a lower furin-mediated cleavage in MERS-CoV S protein expressed by MVA results in S protein that is still maintained in a prefusion state, still allowing S1-specific immune response activation. Since our recent data suggested proper folding and authentic presentation of the trimeric S protein expressed by the MVA vector (15), we hypothesized that processing involving furin-mediated cleavage of the S protein into its membrane-associated S2 subunit and the distal S1 subunit also occurs. Proteolytic cleavage can be followed by shedding of S1, leaving the S2 subunit anchored within the membrane (25). Our results again confirmed the authentic processing of the nonmodified S protein, with prominent S2 expression on the cell surface and obvious S1 shedding.

In previous studies, betacoronavirus S1 shedding had already been observed to inadvertently influence the activation of S-specific antibodies (6, 26, 27). This has also been confirmed for the activation of SARS-CoV-2–neutralizing antibodies after vaccination with the Oxford-AstraZeneca ChAdOx1 nCoV-19 vaccine. The authors discuss that the shedding of cleaved S1 may contribute to a higher proportion of non-neutralizing relative to neutralizing antibodies (28). This is in line with data from a recent study where Barros-Martins and colleagues evaluated the impact of heterologous versus homologous ChAdOx1nCoV-19/BNT162b2 vaccination in humans. Here, individuals who received a homologous BNT162b2 vaccination in a prime-boost schedule showed stronger antibody responses than those receiving homologous ChAdOx1nCoV-19 immunizations (29). Since BNT162b2 is based on a stabilized S protein, we hypothesized that S protein presentation influences immunogenicity, and that S1 dissociation from S2 influences the quantity and quality of MVA-S–activated immune responses. In another study, Dangi and colleagues confirmed that humans vaccinated with a modified S protein exhibited a cross-protective immune response against heterologous coronaviruses (30).

Several approaches have been used to stabilize various class I fusion proteins in their precleaved conformation through structure-based design. For betacoronaviruses it has been suggested that presentation of the S protein in pre- or postfusion conformation has a substantial impact on the ratio of immune responses. In previous studies, 2 proline substitutions at the apex of the central helix and HR1 have been identified that could effectively stabilize MERS-CoV, SARS-CoV, and human coronavirus HKU1 S proteins in the precleaved conformation (27, 31, 32). Such stabilized S proteins were confirmed to be more immunogenic than wild-type S proteins (33, 34).

To evaluate the impact of structural processing of the labile S protein using our vector platform technology, we generated an MVA candidate vaccine expressing precleavage-stabilized S (MVA-ST). Modifications of full-length SARS-CoV-2 S protein expressed by MVA have already been used in other studies (26, 30, 35). As expected, when tested in vivo in mice and hamsters, we observed an improved antibody response after MVA-ST vaccination compared with the original MVA-S candidate vaccine. In line with previous results (36), the general activation of neutralizing antibodies against the Omicron variant was markedly reduced compared with ancestral BavPat1 and Delta variant. Of note, MVA-ST vaccination still induced marginally improved levels of Omicron-neutralizing antibodies compared with MVA-S.

The pattern of antibody responses in MVA-ST–immunized mice clearly exhibited advantageous activation of RBD-, S1-, and S2-binding antibodies. RBD is located in the S1 subunit known as the S protein ectodomain, and both are involved in binding to the specific cellular receptor. Thus both RBD-binding antibodies and those binding S1 elsewhere contribute to efficiently blocking SARS-CoV-2 receptor binding. Moreover, since the fusion peptide region is located within the S2 subunit, S2-binding antibodies are important for inhibiting fusion of the viral and host membranes, which enables release of the viral genome into host cells.

Since coronaviruses can readily generate antibody-escape mutations in the RBD and S1 subunit, activation of antibodies covering the entire S protein is considered desirable to ameliorate vaccine-induced immunity in such events. Indeed, the effectiveness of broadly reactive antibodies has already been confirmed for COVID-19 where REGN-COV2, an antibody cocktail mixture containing 2 neutralizing antibodies targeting the RBD of the SARS-CoV-2 S protein, efficiently reduced viral load in COVID-19 patients (37). Thus, the activation of antibodies targeting different epitopes within the S protein could also be effective against different SARS-CoV-2 VOCs. This was confirmed by our findings that, compared with MVA-S vaccination, superior activation of neutralizing antibodies specific for the S protein of Alpha, Beta, and Gamma variants and against the more recent SARS-CoV-2 variants Delta and Omicron, was achieved in mice and hamsters vaccinated with MVA-ST. The Omicron-characteristic immune evasion is based on a high number of amino acid substitutions present in the RBD. Yet, there is a fraction of broadly reactive antibodies that bind to sites inside and outside the RBD and potently neutralize Omicron (38).

Based on previous studies from other betacoronaviruses, and also from influenza viruses, we hypothesize that this broadly neutralizing capacity may be explained by abundant presentation of the prefusion S2 conformation. S2 has been confirmed to be more highly conserved than S1, and represents a promising antigen to contribute to the induction of broadly protective immunity against current and newly arising coronaviruses (27, 39, 40). In our study, we confirmed more prominent presentation of S2 as a precleavage-stabilized cell-surface protein. This S2 prefusion conformation, in contrast to the postfusion S2 structure, might also contribute to more effectively activating host immune responses (41, 42) and against VOCs harboring high numbers of mutations in S1. We also confirmed the characteristic pattern of S-specific humoral immunity when we comprehensively tested our COVID-19 candidate vaccines in K18-hACE2 mice and the Syrian hamster model. Interestingly, despite these obvious differences in activation of humoral immune responses, the activation of an S1-specific cellular immune response appeared to be comparable following MVA-S and MVA-ST vaccination in mice. In our case, the S1 subunit including the presumed immunodominant SARS-CoV-2 S H2-Kd epitope S_269–278_ is required to induce S1 epitope–specific CD8^+^ T cells (15).

However, since both of the S proteins are initially processed via the *trans*-Golgi network, direct MHC-I presentation should also be efficient in activating CD8^+^ T cell responses specific for S1 epitopes. This is further confirmed by results from Western blot analysis, which detected sufficient and comparable production of S1 antigen in the cell lysates of both MVA-S candidate vaccines. From these data we hypothesize that S1 is properly processed by direct antigen presentation, resulting in sufficient activation of S1-specific CD8^+^ T cells. Here it will be of interest to further characterize levels of S1- and S2-epitope-specific T cells in more detail, especially concerning their role in protective efficacy. A robust activation of S1-epitope-specific T cells should also contribute to a protective immune response against SARS-CoV-2 variants including Omicron that has been confirmed to efficiently evade recognition by many RBD-specific antibodies. Indeed, Omicron-specific CD8^+^ and CD4^+^ T cell responses are well conserved, suggesting negligible immune escape at the level of cellular immunity (43).

Thus, we hypothesize that robust MVA-ST–mediated protection against SARS-CoV-2 variants, including Omicron, will rely on the activation of broadly reactive antibodies targeting conserved antigenic sites within the S protein and the induction of cellular immunity.

Intriguingly, when we evaluated protective efficacy against intranasal SARS-CoV-2 BavPat1 infection, the clinical outcome of both MVA-S– and MVA-ST–vaccinated animals appeared similar, since neither group showed any weight loss or morbidity. Since the primary goal of current SARS-CoV-2 vaccine development and approved vaccines is to prevent symptomatic COVID-19 (2), our results from the infection models indicate that both MVA COVID candidate vaccines are suitable for achieving this. Reduced morbidity is matched by reduced viral loads in the upper and lower respiratory tract of vaccinated animals, although MVA-ST appeared to increase the rate of reduction. One can surmise that significantly reducing SARS-CoV-2 viral load in the lungs results in moderating the severity of the disease. Of note, compared with the MVA-S–vaccinated hamsters, those vaccinated with MVA-ST also showed significantly reduced viral shedding on day 6. This might also be the result of the broader reactive antibody response combined with a robust activation of neutralizing antibodies, leading to rapid virus control in the upper and lower respiratory tract.

Of particular interest, when we characterized the vaccination effect in the hamsters in more detail, postmortem at 6 days after infection, we found that MVA-ST–vaccinated animals seemed more robustly protected from lung pathology, particularly in the alveolar compartment. Diffuse alveolar damage resulting from SARS-CoV-2 BavPat1 infection represents a clinically relevant pathomorphological lesion associated with impaired gas exchange, potentially resulting in acute respiratory distress syndrome. Here, in most of the MVA-ST–vaccinated animals alveolar lesions were completely absent or minimal. In the MVA-S–vaccinated group, the extent of alveolar inflammation and damage was also reduced compared with controls, correlating with the lack of clinical symptoms. However, mild to moderate lesions involving up to 25% to 50% of the lung lobe were still present in all these animals, suggesting incomplete protection of these tissues.

The absence of substantial alveolar pathology and inflammation without any SARS-CoV-2 N antigen expression in the lungs of MVA-ST–vaccinated hamsters favors the idea that the risk of developing long COVID is also reduced. However, this needs to be confirmed in future studies. Since the K18-hACE2 mouse model recapitulates the outcome of severe COVID-19 in humans, efficacy testing of SARS-CoV-2 candidate vaccines in the K18hACE2 SARS-CoV-2 infection model is of substantial value (32, 42). We confirmed the severe and lethal disease outcome in this model for mice that had been vaccinated with nonrecombinant MVA. Despite the absence of death, disease, and even viral load in the lungs of MVA-S–vaccinated mice, substantial pulmonary pathology, including vasculitis and bronchitis, were observed. Of note, vasculitis has been also described as one of the complications of COVID-19 in humans (43).

In contrast, such pathological outcomes were not detected at all in mice vaccinated with MVA-ST. However, since the severity of disease in this model is also mediated by neurological involvement, both the candidate vaccines appeared to readily protect against the lethal outcome of disease presumably through rapidly inhibiting initial replication in the respiratory tract (44). Despite this robust protection achieved in these mice, the observed differences in the outcome of pathology in this model further support the advantage of the modified S protein.

Vice versa, our data also indicate that authentic S processing during viral infection plays an important role in terms of SARS-CoV-2 pathogenesis as an immune evasion strategy. This hypothesis is supported by our results that MVA-S immunogenicity is markedly lower than that of the MVA-ST candidate vaccine. Importantly, we confirmed that a precleavage-stabilized S protein activates a beneficial antibody response. These data suggest that a deeper understanding of the SARS-CoV-2 replication cycle and its potential immune evasion strategies is not only important for better understanding the viral pathogenesis, but also for developing new vaccination strategies.

Taken together, the results show that the availability of a vaccine that not only prevents the obvious development of clinical disease after SARS-CoV-2 infection, but also avoids excessive alveolar damage, inflammation, and subsequent remodeling is highly desirable.

Here, we confirmed the improved efficacy of MVA-ST in preclinical models. These findings merit clinical studies using the MVA-ST candidate vaccine to further characterize the immune responses in humans, not only in homologous immunization cohorts but also in heterologous schedules using mRNA or adenoviral vectors as primary vaccinations. It will also be of particular interest to evaluate how long protective immune responses are maintained and whether broader protection can be achieved. These studies are important due to the still ongoing pandemic and the fact that we still lack data on the impact of vaccine-induced immune-response durability on protection against SARS-CoV-2 infection.

## Methods

### Study design and participants

A phase I clinical trial was conducted to address safety and immunogenicity of the vaccine candidate MVA-S in healthy adults (ClinicalTrials.gov NCT04569383). The study was conducted in Hamburg (Germany) at the University Medical Center Hamburg-Eppendorf (UKE). Study participants were divided into 2 dose groups that received either 1 × 10^7^ IU (low dose) or 1 × 10^8^ IU (high dose) on days 0 and 28 (11).

### Bead-based serological multiplex assay

Serum samples were obtained by venipuncture from vaccinated individuals. Bead-based serological multiplex assay was performed using the MultiCoV-Ab assay validated previously (16, 17). MagPlex Microspheres (Luminex) conjugated to different parts of the S protein based on SARS-CoV-2 Wuhan-Hu-1 reference strain (GenBank accession no. MN908947.3) were used: purified trimeric S protein, S1 domain, RBD (all produced in-house), and S2 domain (Sino Biological). Serum samples were incubated at a dilution of 1:400 for 2 hours at room temperature. Subsequently, the beads were washed using 100 μL of washing buffer (PBS supplemented with 0.05% [v/v] Tween 20) per well with the aid of a LifeSep magnetic separator unit (Dexter Magnetic Technologies). After 3 washing steps, bound antibodies were detected using PE-coupled secondary anti–human IgG antibodies (Dianova, 109-116-098, lot 148837; 3 μg/mL), incubated for 45 minutes at room temperature. Samples were measured using the Bio-Plex 200 System (Bio-Rad Laboratories), controlled by BioPlex manager software, version 5.0.0.531. Cutoff samples with a known MFI value were generated as previously established (44) and included on each plate as quality control.

### Immunofluorescent staining and confocal microscopy

To quantify the cellular localization of S1 and S2, Huh-7 cells were infected with MVA-S or -ST (MOI 0.5) or transfected with plasmids encoding nonstabilized S protein. Eighteen hours after transfection/infection, S located at the cell surface was labeled at 4°C prior to fixation using a human-derived anti-S1 monoclonal antibody (generated and provided by F. Klein, Institute of Virology, University Hospital of Cologne, Germany; ref. 18). Subsequently, cells were fixed with 4% paraformaldehyde, permeabilized with 0.1% Triton X-100, and total S was labeled using anti-S2 antibody from mouse (GeneTex, GTX632604, clone 1A9; 1:100). Polyclonal goat anti-mouse–Alexa Fluor 594 (catalog A-11005) and goat anti-human–Alexa Fluor 488 (catalog A-48276) secondary antibodies (Thermo Fisher Scientific; 1:200) were used to visualize S-specific staining by fluorescence. Nuclei were stained with 1 μg/mL DAPI (Sigma-Aldrich, D9542) and cells were analyzed using the Leica SP2 confocal microscope (Leica) with ×63 objective. All quantification of immunofluorescence-related data was performed with ImageJ/Fiji v.1.51 (45). To quantify surface S, optical sections of Huh-7 cells (500 nm/slice) were acquired in order to project the entire cell. Pixel intensities were measured in S1 (surface) and in S2 (total S) channels. The ratio between S1 and S2 values was calculated to yield the relative surface expression. Prior to each analysis, cell borders were determined using standard selection tools.

### SARS-CoV-2 S1 surface staining for flow cytometry

A549 cells were infected with 1 MOI MVA-S/-ST and MVA and incubated for 16 hours at 37°C, and cells were harvested and plated onto 96-well U-bottom plates at 2 × 10^5^ cells/well. Cells were incubated with purified anti–mouse CD16/CD32 (Fc block; BioLegend, clone 93; 1:500) for 15 minutes on ice. Cells were incubated with anti-S1 human monoclonal antibody (see above) for 30 minutes on ice and then with goat anti–human IgG (H+L)–Alexa Fluor 488 (Thermo Fisher Scientific, A-48276; 1:3000) for 30 minutes on ice. Cells were then stained with fixable dead cell viability dye Zombie Aqua (BioLegend, 423101; 1:1000). After staining, cells were fixed using Fixation Buffer (BioLegend) according to the manufacturer’s protocol. Data were acquired using the MACSQuant VYB Flow Analyzer (Miltenyi Biotec) and analyzed using FlowJo (FlowJo LLC, BD Life Sciences).

### PRNT50

Serum samples were used to analyze neutralization capacity against SARS-CoV-2 (isolate Germany/BavPat1/2020; isolate hCoV-19/USA/PHC658/2021, lineage B.1.617.2 Delta variant; isolate hCoV-19/USA/MD-HP20874/2021, lineage B.1.1.529, Omicron variant) received from BEI Resources, NIAID, NIH, as previously described with some modifications (46). Heat-inactivated serum samples were serially diluted 2-fold in duplicate in 50 μL DMEM. Then, 50 μL of virus suspension (600 TCID_50_) was added to each well and incubated at 37°C for 1 hour before placing the mixtures on Vero E6 cells (ATCC, CRL-1586), seeded in 96-well plates. After incubation for 45 minutes, 100 μL of a 1:1 mixture of prewarmed DMEM and Avicel RC-591 (Dupont, Nutrition & Biosciences) was added and plates were incubated for 24 hours. After incubation, cells were fixed with 4% formaldehyde/PBS and stained with a polyclonal rabbit antibody against SARS-CoV-2 nucleoprotein (Sino Biological, 40588-T62; 1:2000) and a secondary HRP-labeled goat anti–rabbit IgG (Agilent Dako, P044801-2; 1:1000). The signal was developed using a precipitate-forming TMB substrate (True Blue, KPL SeraCare, 5510-0030) and the number of infected cells per well was counted by using an ImmunoSpot reader (CTL Europe GmbH). The reciprocal of the highest serum dilution allowing reduction of greater than 50% plaque formation was calculated as the serum neutralization titer (PRNT_50_) using the BioSpot Software Suite (CTL Europe GmbH).

### SARS-CoV-2 VNT100

The neutralizing activity of mouse serum antibodies was investigated based on a previously published protocol (47). Briefly, samples were serially diluted in 96-well plates starting from a 1:16 serum dilution. Samples were incubated for 1 hour at 37°C together with 100 PFU of SARS-CoV-2. Cytopathic effects on Vero cells were analyzed 4 days (BavPat1, Alpha, Gamma) or 6 days (Zeta) after infection. Neutralization was defined as absence of the cytopathic effects compared with virus controls. For each test, a positive control (human monoclonal antibody; refs. 18, 48) was used in quadruplicate as an interassay neutralization standard.

### Challenge-infection experiments in Syrian hamsters and K18-hACE2 mice

For SARS-CoV-2 challenge infection, animals were kept in individually ventilated cages (IVCs, Tecniplast) in approved BSL-3 facilities. All animal and laboratory work with infectious SARS-CoV-2 was performed in a BSL-3e laboratory and facilities at the Research Center for Emerging Infections and Zoonoses (RIZ), University of Veterinary Medicine, Hanover, Germany.

All animals were infected under anesthesia via the intranasal route with 1 × 10^4^ (hamsters) or 3.6 × 10^4^ (mice) TCID_50_ of SARS-CoV-2 (isolate Germany/BavPat1/2020, NR-52370) received from BEI Resources, NIAID, NIH. After challenge infection, hamsters and mice were monitored at least twice daily for well being, health constitution, and clinical signs using a clinical score sheet (Supplemental Table 2). Body weights were checked daily.

### Viruses

SARS-CoV-2 (isolate Germany/BavPat1/2020, NR-52370; isolate hCoV-19/USA/PHC658/2021, lineage B.1.617.2 Delta variant, NR-55611; isolate hCoV-19/USA/MD-HP20874/2021, lineage B.1.1.529, Omicron variant, NR-56461) received from BEI Resources, NIAID, NIH, were propagated in Vero cells in DMEM (Sigma-Aldrich) supplemented with 2% FBS, 1% penicillin-streptomycin, and 1% L-glutamine at 37°C. All infection experiments with SARS-CoV-2 were performed in BSL-3 laboratories at the RIZ, University of Veterinary Medicine Hannover, Germany or the Institute of Virology, Philipps University Marburg, Germany.

### Measurement of viral burden

Lung tissue samples of immunized and challenged hamsters or mice excised from the right lung lobes, and brain tissue excised from the right brain of mice were homogenized in 1 mL DMEM containing antibiotics (penicillin and streptomycin, Gibco). Tissue was homogenized using the TissueLyser-II (Qiagen), and aliquots were stored at –80°C. Viral titers were determined on Vero cells as median TCID_50_ units. Briefly, Vero cells were seeded in 96-well plates and serial 10-fold dilutions of homogenized lung samples in DMEM containing 5% FBS. After incubation for 96 hours at 37°C, cytopathic effects in Vero cells were evaluated and calculated as TCID_50_ unit per gram or mL using the Reed-Muench method. For samples without cytopathic effect, data points were set to half of the detection limit for statistical analysis purposes.

### Statistics

Data were prepared using GraphPad Prism 9.0.0 and R 4.2.1 (https://cran.r-project.org/) and expressed as mean ± standard error of the mean (SEM) or median ± interquartile range. Data were analyzed by 1-way ANOVA and Kruskal-Wallis test to compare 3 or more groups. A *P* value of less than 0.05 was used as the threshold for statistical significance.

### Study approval

#### Human specimens.

The study design was reviewed and approved by the Competent National Authority (Paul-Ehrlich-Institut [PEI], Langen, Germany) and the Ethics Committee of the Hamburg Medical Association. The study was performed in accordance with the Declaration of Helsinki in its version of Fortaleza 2013 and ICH-GCP. All participants voluntarily enrolled in the study by signing an informed consent form after receiving detailed information about the clinical study.

#### Hamster and mouse studies.

All animal experiments including SARS-CoV-2 infection under BSL-3 conditions were handled in compliance with the European and national regulations for animal experimentation (European Directive 2010/63/EU; Animal Welfare Acts in Germany) and Animal Welfare Act, approved by the Regierung von Oberbayern (Munich, Germany) and the Niedersächsisches Landesamt für Verbraucherschutz und Lebensmittelsicherheit (LAVES, Lower Saxony, Germany).

## Author contributions

AV and GS conceptualized the study and revised the manuscript. AT constructed and characterized the vaccines, and performed experiments for safety and immunogenicity in BALB/c mice together with JHS, LL, GK, KB, SJ, and A Freudenstein. CMZN established in vivo SARS-CoV-2 infection models, performed in vitro and in vivo experiments to characterize safety, immunogenicity, and efficacy together with DLS, TT, SC, LMS, and AV. CR, SH, AK, and SB characterized S protein expression and VNT_100_. CD, A Fathi, MLW, LM, and MMA provided human sera from the phase I trial. RF, MK, ME, NSM, AD, and PK analyzed human sera. MC, FA, GB, and WB provided pathological investigations. CMZN and AT performed experiments, acquired data, interpreted data, and revised the manuscript. CMZN, AT, AV, and GS wrote the manuscript together with all coauthors.

## Supplementary Material

Supplemental data

## Version 1 10/27/2022

In-Press Preview

## Version 2 12/15/2022

Electronic publication
