# Supplementary material for: Stabilized recombinant SARS-CoV-2 spike antigen enhances vaccine immunogenicity and protective capacity
Source: J Clin Invest. 2022 Dec 15;132(24):e159895. doi: 10.1172/JCI159895 (PMC9754005; doi:10.1172/JCI159895)
Supplement: Supplemental data [file jci-132-159895-s033.pdf]

Supplemental Methods, Tables, and Figures

Supplement to:

Stabilized recombinant SARS-CoV-2 spike antigen enhances vaccine immunogenicity and protective capacity

Supplemental Materials & Methods

Cell cultures.

DF-1 cells (ATCC® CRL-12203™) were maintained in VP-SFM medium (Thermo Fisher Scientific), 2% heat-inactivated fetal bovine serum (FBS) (Thermo Fisher Scientific) and 2% L-glutamine (Thermo Fisher Scientific). Primary chicken embryonic fibroblasts (CEF) were prepared from 10 to 11-day-old chicken embryos (SPF eggs, VALO) using recombinant trypsin (Tryple TM, Thermo Fisher Scientific) and maintained in VP-SFM medium, 10% FBS and 1% L-glutamine. Vero cells (ATCC® CCL-81™), Vero E6 cells (ATCC® CRL-1586™), Human HaCat cells and Huh7 cells (CLS Cell Lines Service GmbH) were maintained in Dulbecco's Modified Eagle's Medium (DMEM), 10% FBS and 1% MEM non-essential amino acid solution (Sigma-Aldrich). Human A549 cells (ATCC® CCL-185™, LGC standards) were maintained in DMEM with high glucose and 10% FBS. Human HeLa cells (ATCC® CCL-2) were maintained in Minimum Essential Medium Eagle (MEM) (Sigma-Aldrich), 7% FBS and 1% MEM non-essential amino acid solution.

Plasmid construction.

The coding sequence of the full-length SARS-CoV-2 S protein was modified in silico by introducing silent mutations to remove runs of guanines or cytosines and termination signals of

vaccinia virus-specific early transcription. Five mutations (R682G, R683S, R685S, K986P and V987P) were introduced to obtain the pre-cleaved stabilized spike sequence (SARS-2-ST). The modified SARS-2-ST cDNA was produced by DNA synthesis (Eurofins) and cloned into the MVA transfer plasmid pIIIH5red under transcriptional control of the synthetic vaccinia virus early/late promoter PmH5 to obtain the MVA expression plasmid pIIIH5red-SARS-2-ST.

Generation and characterization of the candidate vaccine MVA-SARS-2-ST.

To achieve stabilization of the SARS-2 spike protein, we inactivated the furin cleavage site (RRAR682–685GSAS) and added proline substitutions in the loop between the first heptad repeat (HR1) and the central helix (K986P, V987P) as previously established (1, 2) (Supplemental Figure 1A). cDNA containing the optimized gene sequence encoding for stabilized SARS-CoV-2-S (SARS-2-ST) from the virus isolate Wuhan HU-1 (GenBank accession no. MN908947.1) (Supplemental Figure 1B) was placed under the transcriptional control of the enhanced synthetic vaccinia virus early/late promoter PmH5 in the MVA vector plasmid pIIIH5red-SARS-2-ST, and introduced by homologous recombination into deletion site III in the MVA genome (Supplemental Figure 1C). MVA (clonal isolate MVA-F6-sfMR) was grown on CEF under serum-free conditions and served a as non-recombinant backbone virus to construct MVA vector viruses expressing the SARS-CoV-2-ST gene sequences. Briefly, monolayers of 90-95% confluent DF-1 cells were grown in six-well tissue culture plates (Sarstedt), infected with non-recombinant MVA at 0.05 multiplicity of infection (MOI), and transfected with plasmid pIIIH5red-SARS-2-ST DNA using X-tremeGENE HP DNA Transfection Reagent (Roche Diagnostics) according to the manual. Afterwards, cell cultures were collected and recombinant MVA viruses were clonally isolated by serial rounds of plaque purification on DF-1 cell monolayers monitoring for transient co-production of the red fluorescent marker protein mCherry. To obtain vaccine preparations, the resulting recombinant MVA-SARS-2-ST (MVA-ST) was amplified on DF-1 cell monolayers grown in T175 tissue

culture flasks, purified by ultracentrifugation through 36% sucrose and reconstituted to high titer stock preparations in Tris-buffered saline pH 7.4. Plaque-forming units (PFU) were counted to determine viral titers. The virus was purified and quality controlled according to standard procedures for generating recombinant MVA vaccines (3). During repetitive plaque purification using transient coproduction of the fluorescent marker protein mCherry to screen for red fluorescent cell foci, we analyzed the genetic integrity and stability by PCR using oligonucleotide primers to confirm MVA identity (PCR of the six major deletion sites of MVA in MVA-ST; Supplemental Figure 1, D-F) and correct insertion of stabilized S gene sequences within the MVA deletion site III (Supplemental Figure 1G). To evaluate growth behavior, we infected permissive DF-1 cells and non-permissive human cells (HaCat, HeLa and A549) and analyzed viral load as established before. As expected, recombinant MVA-ST replicated efficiently in the avian cell line (DF-1) but not in cells of mammalian origin (HaCat, HeLa and A549) (Supplemental Figure 2).

Western blot analysis of recombinant protein.

DF-1 cells were infected at MOI 10 with recombinant or non-recombinant MVA or remained uninfected (mock). At indicated time points of infection, cell lysates were prepared from infected cells and stored at -80°C. Proteins from lysates were separated by electrophoresis on a SDS-10% polyacrylamide gel (SDS-PAGE, Bio-Rad) and subsequently transferred to a nitrocellulose membrane by electroblotting. The blots were blocked in a phosphate buffered saline (PBS) buffer containing 5% nonfat dried milk powder (PanReac AppliChem) and 0.1% Tween-20 (Sigma-Aldrich) and incubated overnight with primary antibodies targeting S1 (Genetex, Cat-No. GTX635654, clone HL6, 1:2000) or S2-domain (GeneTex, Cat-No. GTX632604, clone 1A9, 1:4000). Next, membranes were washed with 0.1% Tween-20 in PBS and incubated with anti-mouse (Agilent Dako, Cat-No. P044701-2, 1:5000) or anti-rabbit IgG (Cell Signaling, Cat-No. 7074, 1:5000), conjugated to horseradish peroxidase. Blots were

washed and developed using SuperSignal® West Dura Extended Duration substrate (Thermo Fisher Scientific, Cat-No. 34075) in ChemiDoc MP Imaging System (Bio-Rad).

Immunofluorescence staining of recombinant SARS-2-ST protein.

To confirm S protein cell surface expression and trafficking, Vero cells were infected with 0.05 MOI MVA-SARS-2-S/2-ST or MVA and incubated at 37°C. After 24 h, cells were fixed with 4% paraformaldehyde (PFA) for 10 min on ice, washed two times with PBS, and when indicated permeabilized with 0.1% Triton X-100 (Sigma-Aldrich) in PBS. Cells were stained with a mouse monoclonal antibody obtained against the S2 protein (SARS-1-S, GeneTex, Cat-No. GTX632604, clone 1A9, 1:200) before fixation with PFA. Polyclonal goat anti-mouse secondary antibody (Life Technologies, Cat-No. A-11017, 1:1000) was used to visualize S2-specific staining by green fluorescence. Nuclei were stained with 1 µg/ml of 4,6-diamidino-2-phenylindole (DAPI) (Sigma-Aldrich, Cat-No. D9542) and cells were analyzed using the Keyence BZ-X700 microscope (Keyence) with a ×100 objective.

Vaccination experiments in mice and hamsters.

Female BALB/c mice (BALB/cAnNCrl, inbred, 6 to 10 weeks old) were purchased from Charles River Laboratories (Sulzfeld, Germany). Male Syrian hamsters (10 week-old, Mesocricetus auratus; breed RjHan:AURA) were purchased from Janvier Labs (Saint Berthevin, France). K18-hACE2 mice (034860-B6.Cg-Tg(K18-ACE2)2Prlman/J, on the C57BL/6 background, 6-8 weeks old, 50:50 males:females) were purchased from The Jackson Laboratory (Bar Harbor, USA). Hamsters and mice were maintained under specified pathogen-free conditions, had free access to food and water, and were allowed to adapt to the facilities for at least one week before vaccination experiments were performed. Immunizations were performed using intramuscular applications with vaccine suspension containing 10^8 PFU recombinant MVA-SARS-2-S/2-ST, MVA or PBS (mock) into the quadriceps muscle of the left hind leg. Boost vaccinations were performed 21 days later.

Blood was collected on days 0, 18, 35 for the BALB/c mice, on days 0, 21, 42 and 55 for the hamsters and on days 0, 18, 31, 55 or 57 for the K18-hACE mice. Coagulated blood was centrifuged at 1300×g for 5 min in serum tubes (Sarstedt AG&Co.) to separate serum, which was stored at -80 °C until further analysis.

Ethics statement.

All animal experiments were handled in compliance with the European and national regulations for animal experimentation (European Directive 2010/63/EU; Animal Welfare Acts in Germany) and Animal Welfare Act, approved by the Regierung von Oberbayern (Munich, Germany) and by the Niedersächsisches Landesamt für Verbraucherschutz und Lebensmittelsicherheit (LAVES, Lower Saxony, Germany).

Antigen-specific IgG ELISA.

SARS-2-S-specific serum IgG titers were measured by ELISA as described previously (4). ELISA plates (Nunc MaxiSorp Plates, Thermo Fisher Scientific) were coated with 50 ng/well recombinant SARS-CoV-2 spike, S1, S2 and receptor binding domain (RBD) protein (from AcroBiosystems and The Native Antigen Company, Supplemental Table 1) overnight at 4°C. Plates were washed and then blocked with blocking buffer containing 1% BSA and 0.15 M sucrose (both Sigma-Aldrich) dissolved in PBS. Plates were then probed with sera serially diluted 3-fold in PBS containing 1% BSA (PBS/BSA), starting at a dilution of 1:100, and then probed with goat anti-mouse IgG HRP (Agilent Dako, Cat-No. P044701-2, 1:2000) or goat anti-hamster IgG (H+L)-HRP (SouthernBiotech, Cat-No. 6060-05, 1:6000) diluted in PBS/BSA. Bound antibody was visualized by adding 3´3´, 5´5´-Tetramethylbenzidine (TMB) Liquid Substrate System for ELISA (Sigma-Aldrich) followed by Stop Reagent for TMB Substrate (450 nm, Sigma-Aldrich) after a color change was observed. The absorbance was measured at 450 nm with a 620 nm reference wavelength using the Sunrise™ microplate reader (Tecan Trading AG). ELISA data were normalized using the positive control. The cut-off value

for positive mouse serum samples was determined by calculating the mean of the normalized OD 450 nm values of the PBS control group sera plus 6 standard deviations (mean + 6 SD).

T cell analysis by Enzyme-Linked Immunospot (ELISPOT).

At day 14 post prime-boost vaccination, mice were sacrificed and splenocytes were prepared. Splenocytes were washed and resuspended in RPMI-10 (Sigma-Aldrich). ELISPOT assays (Mabtech ELISpot kit for mouse IFN-γ, Biozol) were performed following the manufacturer's instructions. Briefly, $2x10^5$ splenocytes/100 µl were seeded in 96-well plates and stimulated with individual peptides (2 µg/ml RPMI-10). Non-stimulated cells and cells stimulated with phorbol myristate acetate (PMA) /Ionomycin (Sigma-Aldrich) or vaccinia virus peptide SPGAAGYD ($F2(G)_{26\text{-}34}$; H-$2L^d$; (5)) served as controls. After 37°C for 48 h, plates were stained according to the manufacturer's instructions. Spots were counted and analyzed using an automated ELISPOT plate reader and software (A.EL.VIS Eli.Scan, A.EL.VIS ELISPOTAnalysis Software, Hannover, Germany).

T cell analysis by Intracellular Cytokine Staining (ICS).

ICS methods were described previously (4). Briefly, splenocytes were diluted in RPMI-10 and plated onto 96-well U-bottom plates using 10^6 cells/well. Cells were stimulated with 8 µg/ml $S_{268\text{-}276}$ (GYLQPRTFL) peptide or vaccinia virus peptide $F2(G)_{26\text{-}34}$ (SPGAAGYD) to analyze SARS-2-S- or MVA-specific CD8+ T cells. PMA plus ionomycin served as positive controls and RPMI alone was used as a negative control. After 2 h at 37°C, brefeldin A (Biolegend) was added according to the manufacturer's instructions and stimulated cells were further maintained for 4 h at 37°C. After stimulation, cells were stained extracellularly with anti-mouse CD3 phycoerithrin (PE)-Cy7 (Biolegend, clone 17A2, 1:100), anti-mouse CD4 Brilliant Violet 421 (Biolegend, clone GK1.5, 1:600), anti-mouse CD8α Alexa Fluor 488 (Biolegend, clone 53-6.8, 1:300), and purified CD16/CD32 (Fc block; Biolegend, clone 93, 1:500). Cells were then stained with the fixable dead cell viability dye Zombie Aqua (Biolegend, Cat-No. 423101,

1:800) and fixed using Fixation Buffer (Biolegend) and permeabilised using Intracellular Staining Permeabilization Wash Buffer (Biolegend) according to the manufacturer's protocol. Samples were stained intracellularly with anti-mouse IFN-γ (Biolegend, clone XMG1.2, 1:200) plus anti-mouse TNF-α (Biolegend, clone MP6-XT22, 1:200). Data was acquired by the MACSQuant VYB Flow Analyser (Miltenyi Biotec) and analyzed using FlowJo (FlowJo LLC, BD Life Sciences).

Real-time PCR to detect SARS-CoV-2.

RNA was extracted from lung tissue samples using Qiamp Viral RNA Mini Kit (Qiagen) and eluted in 30 µl of RNase-free water (Sigma GmbH) according to the manufacturer's protocol. For SARS-CoV-2 RNA amplification, the commercially available AgPath-IDTM One-Step RT-PCR kit (Thermo Fisher Scientific) in a CFX96-Touch Real-Time PCR system (Bio-Rad) was used. The RT-qPCR assay specific for the RdRp gene of SARS-CoV-2 and recommended by the WHO, were used: SARS-2-IP4, forward primer (5′- GGT AAC TGG TAT GAT TTC G -3'), reverse primer (5′- CTG GTC AAG GTT AAT ATA GG-3') and probe (5'-TCA TAC AAA CCA CGC CAG G-3' [5']FAM [3']BHQ-1)]. The PCR program included reverse transcription at 50˚C for 20 min; denaturation at 95˚C for 10 min; 50 cycles of 95˚C for 15 sec (denaturation), and 58˚C for 45 sec (annealing and elongation). The relative fluorescence units (RFU) were measured at the end of the elongation step. The sample Ct value was correlated to a standard RNA transcript and the quantity of viral RdRp copy numbers per ul of total RNA was calculated.

Histological evaluation of lung pathology in hamsters and mice.

Left lung lobes were fixed by instillation and immersion in 10% buffered formalin (6). Tissues were subsequently embedded in paraffin and cut into 2-3 µm thick sections. Lesions were evaluated on hematoxylin and eosin (HE) stained sections in a blinded fashion with a semiquantitative scoring system. Briefly, the evaluation included assessment of alveolar lesions

(inflammation, regeneration, necrosis/desquamation and loss of alveolar cells, atypical large/syncytial cells, intraalveolar fibrin, alveolar edema, hemorrhage), airway lesions (inflammation, necrosis, hyperplasia) and vascular lesions (vasculitis, perivascular cuffing, edema, and hemorrhage). The total scores reflect the sum of all scores in the respective category. Details on the scoring system have been described previously (7).

Immunohistochemistry to detect SARS-CoV-2 antigen.

Detection of SARS-CoV-2 nucleocapsid protein was performed on formalin-fixed, paraffin-embedded lung tissue using a monoclonal mouse antibody (Sino Biological, Cat-No. 40143-MM05) and the Dako EnVision+ polymer system (Dako Agilent Pathology Solutions) as described previously (7). Evaluation was performed semiquantitatively (0 = no antigen; 1 = minimal, single foci, less than 1%; 2 = mild, 2-25%; 3 = moderate, 26-50%; 4 = severe, 51-75%; 5 = subtotal, >75% of tissue affected).

Supplemental Table 1. Recombinant SARS-CoV-2 S proteins used for ELISA.

Recombinant Protein	Company	Catalogue No.
SARS-CoV-2 S protein, His Tag	ACROBiosystems	SPN-C52H4
SARS-CoV-2 (COVID-19) S1 protein, His Tag	ACROBiosystems	S1N-C52H3
SARS-CoV-2 Spike Glyoprotein (S2), Sheep Fc-Tag	The Native Antigen Company	REC31807-500
SARS-CoV-2 (COVID-19) S protein RBD, His Tag	ACROBiosystems	SPD-C52H3

Supplemental Table 2. Clinical Scores used for evaluation of SARS-CoV-2 infected animals.

**Clinical Scores**	**Body weight**	**Cardiovascular system**	**Fur/ skin condition**	**Lower respiratory tract**	**Upper respiratory tract**	**Environment**	**Social behaviour/ general condition/ locomotion**	**Neurological scoring**
**Symptoms (mild to moderate to severe)**	<5% weight loss	normal	normal fur	normal	mild serous ocular or nasal discharge	normal	normal stimulus response (attentive, curious)	normal
	5-10% weight loss	reduced skin turgor/ mild enophthalmia ("sunken eyes")/ mild dehydration	slightly ruffled fur	mild tachypnoea (low abdominal respiration)	moderate serous to mucous ocular or nasal discharge	Rummaged beeding	suppressed eating, drinking, or running, (tired) or nervous/ hyperactive or lack of nesting behaviour	reduced physical activity
	11-15% weight loss	moderate to severe dehydration/ moderate to severe enophthalmia ("sunken eyes")	ruffled fur	moderate tachypnoea (significant abdominal respiration)	moderate mucous to purulent ocular or nasal discharge	soft feces	unusual behaviour, fearful, hiding in houses	mild neurological symptoms
	16-<20% weight loss	reduced body temperature, acrocyanosis	ruffled fur, puffy appearance and piloerection	dyspnoea (significant)	severe mucous to purulent ocular or nasal discharge	diarrhea	depressed, reduced interaction with other animals/ apathetic, slowed stimulus response	moderate neurological symptoms,
	acute weight loss ≥20% compared to initial weight (≤24h)	circulatory shock	severe/ extensive lesions, severe skin inflammation	cold pale mucosa	swollen eye area, eye and nose openings clotted by secretion, or purulent inflammations of the connective tissue of the eye	hemorrhagic diarrhea	apathy/ lethargy	ataxia, paralysis, tremors, convulsions, circle walking

Supplemental References

1. Pallesen J, et al. Immunogenicity and structures of a rationally designed prefusion MERS-CoV spike antigen. *Proc Natl Acad Sci U S A.* 2017;114(35):E7348-e57.
2. Bos R, et al. Ad26 vector-based COVID-19 vaccine encoding a prefusion-stabilized SARS-CoV-2 Spike immunogen induces potent humoral and cellular immune responses. *NPJ Vaccines.* 2020;5:91.
3. Kremer M, et al. Easy and efficient protocols for working with recombinant vaccinia virus MVA. *Methods Mol Biol.* 2012;890:59-92.
4. Tscherne A, et al. Immunogenicity and efficacy of the COVID-19 candidate vector vaccine MVA-SARS-2-S in preclinical vaccination. *Proc Natl Acad Sci U S A.* 2021;118(28).
5. Tscharke DC, et al. Poxvirus CD8+ T-cell determinants and cross-reactivity in BALB/c mice. *J Virol.* 2006;80(13):6318.
6. Meyerholz DK, et al. Approaches to Evaluate Lung Inflammation in Translational Research. *Vet Pathol.* 2018;55(1):42-52.
7. Bošnjak B, et al. Intranasal Delivery of MVA Vector Vaccine Induces Effective Pulmonary Immunity Against SARS-CoV-2 in Rodents. *Front Immunol.* 2021;12:772240.

A

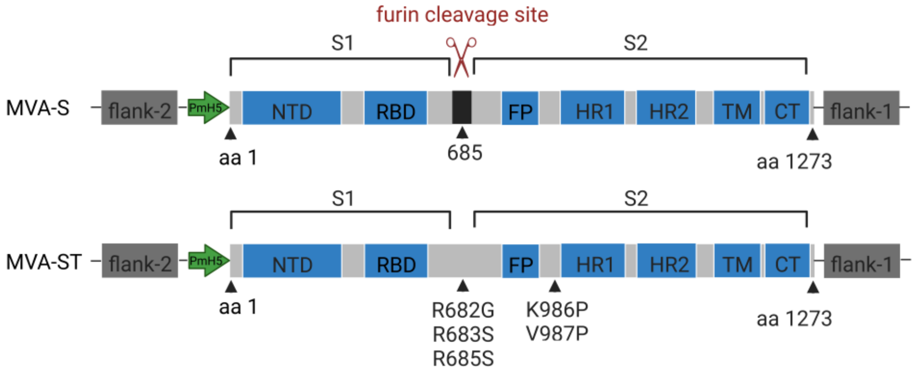

Supplemental Figure 1. Construction and characterization of MVA-S and MVA-ST. (**A**) Schematic representation of the recombinant spike proteins expressed by MVA-S or MVA-ST; **NTD**, N-terminal domain; **RBD**, receptor binding domain; **FP**, fusion peptide; **HR1**, heptad repeat 1; **HR2**, heptad repeat 2; **TM**, transmembrane domain; **CT**, C-terminal domain. MVA-S produces the native SARS-CoV-2 spike protein containing the furin cleavage site for proteolytical processing of the S1 and S2 subdomains. MVA-ST encodes for the same SARS-CoV-2-S protein but contains the five indicated amino acid exchanges for pre-fusion stabilization. Created with BioRender.com.

B

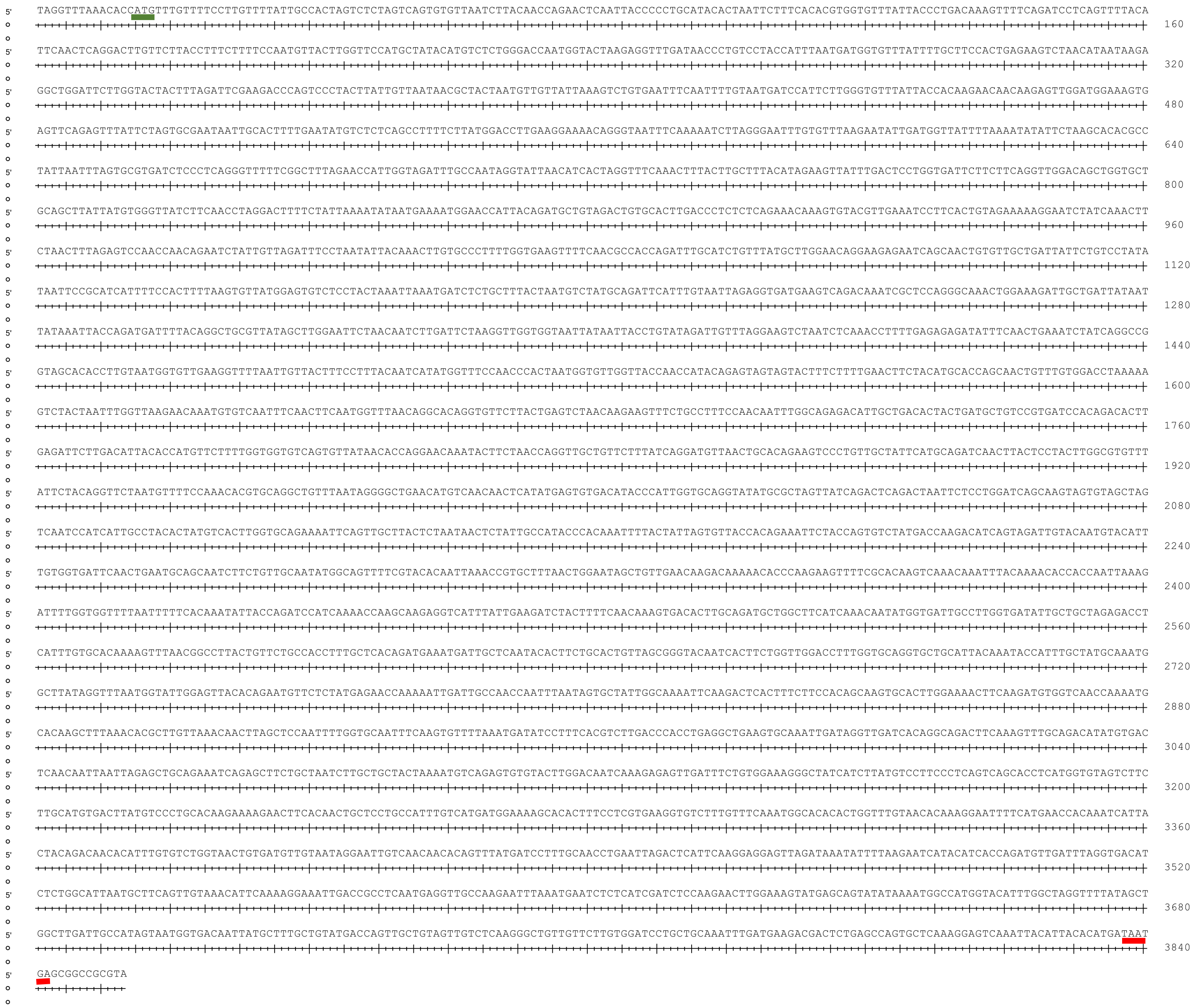

(**B**) DNA sequence of the modified SARS-2-ST. Green line shows the sequence for start signal (ATG) and red line shows the sequence for the stop signal (TAATGA).

C

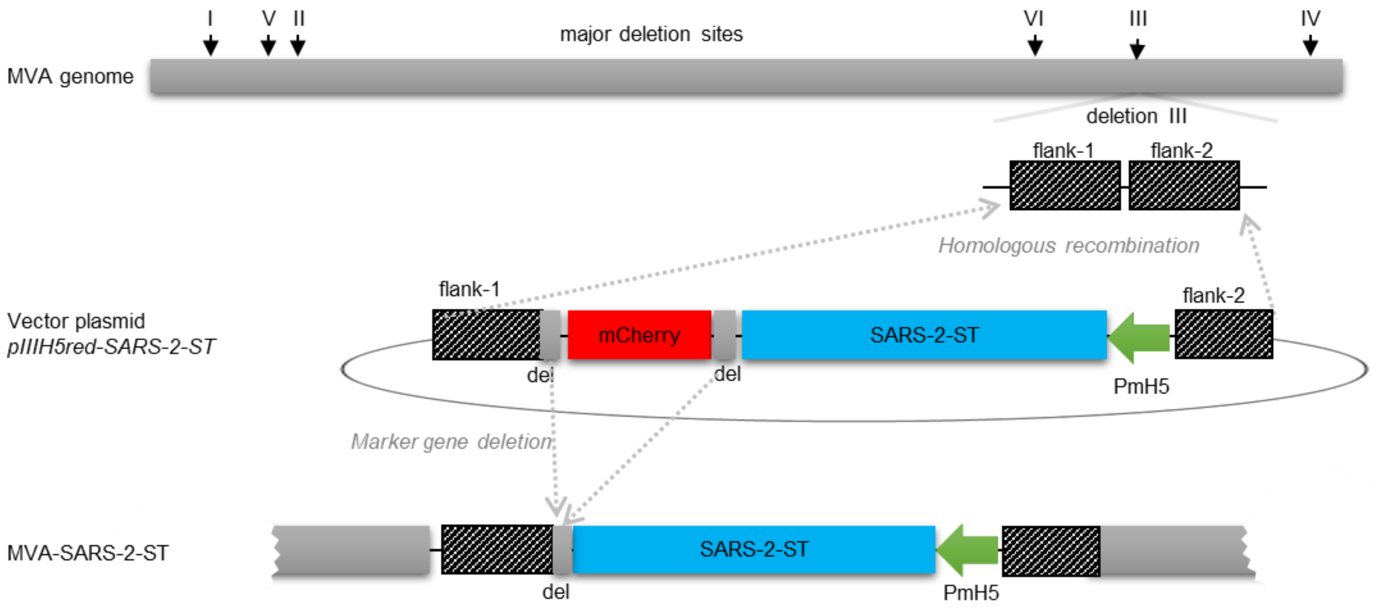

(**C**) Schematic diagram of the MVA genome with the major deletion sites I to VI. A modified version of the spike protein of SARS-CoV-2 isolate Wuhan-HU-1 (SARS-2-ST) was inserted into deletion III and placed under transcriptional control of the vaccinia virus promoter PmH5. Insertion occurred via homologous recombination between MVA DNA sequences (flank-1 and flank-2) adjacent to deletion site III in the MVA genome and copies cloned in the vector plasmid. MVA-ST was isolated by plaque purification screening for co-production of the red fluorescent marker protein mCherry. A repetition of short flank-1 derived DNA sequences (del) served to remove the marker gene by intragenomic homologous recombination (marker gene deletion).

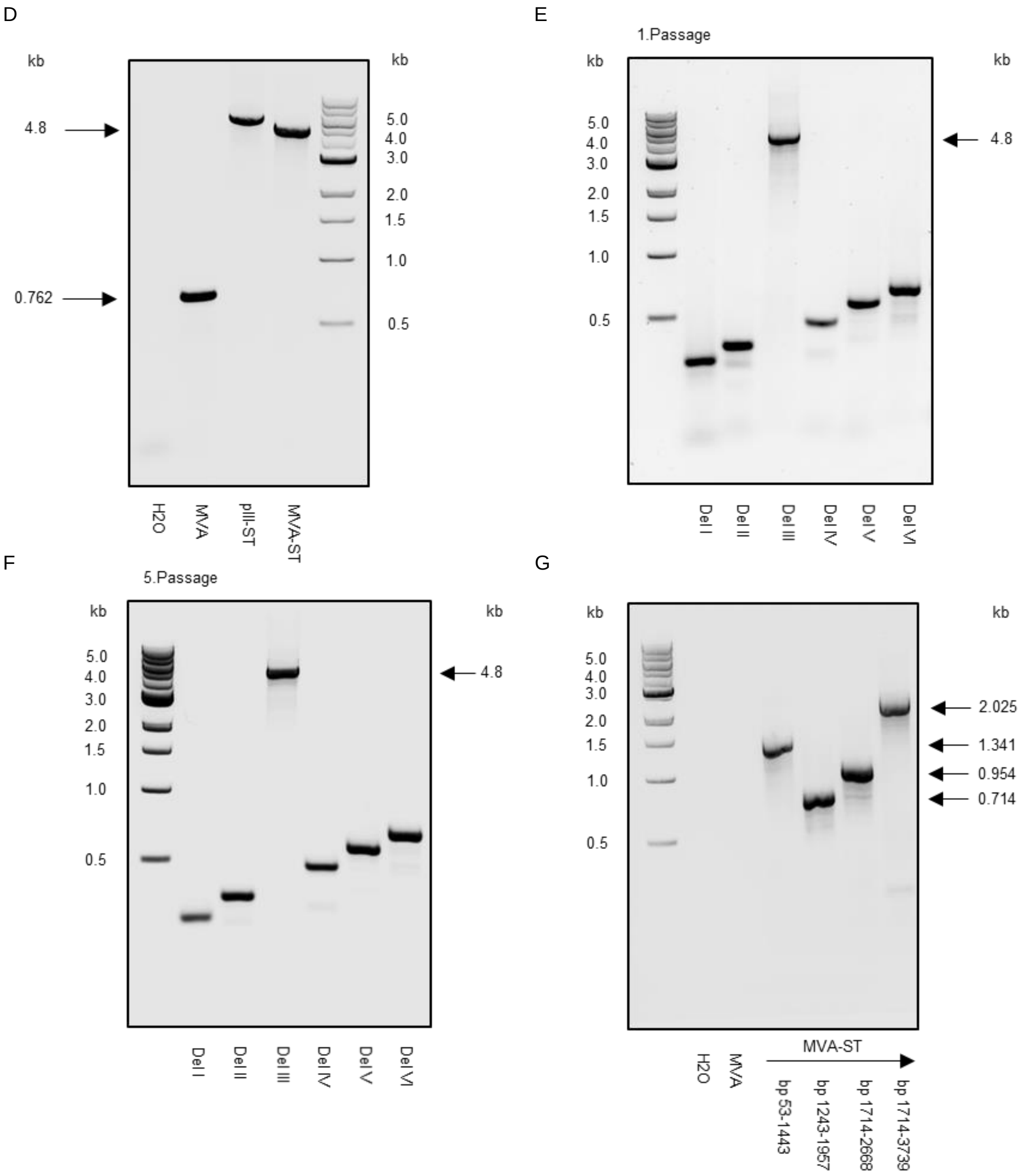

(**D**) Genetic integrity of MVA-ST. PCR analysis of genomic viral DNA confirmed the stable insertion of the SARS-2-ST sequence into deletion site III of the MVA genome. The precise intragenomic deletion of the marker gene mCherry during plaque purification was revealed by amplification of a PCR product with the expected molecular weight (4.8 kb) from MVA-ST genomic DNA compared to pIIIH5red-SARS-2-ST plasmid DNA template (pIII-ST). From the deletion III site-specific oligonucleotide primers allowed for amplification of a characteristic 0.762 kb DNA fragment from genomic DNA of non-recombinant MVA. (**E** and **F**) Genetic stability of MVA-ST after serial growth amplification in DF-1 cell cultures. DF-1 cells were infected with MVA-ST at MOI of 0.05 and incubated for 48 h. Subsequently, the amplified virus was harvested and used to re-infect fresh DF-1 cells at MOI of 0.05 for 48 h. This procedure was performed five times. MVA-ST genetic stability was tested by PCR analysis of genomic viral DNA and monitored for recombinant gene expression by S-specific immunostaining. PCR analysis demonstrated genetic stability for six loci in the MVA-ST genome (deletion sites Del I-VI) including the heterologous SARS-2-ST gene sequences inserted into the site of deletion III (Del III) with the amplification of characteristic size DNA fragments from viral DNA prepared after the first or fifth round of MVA-ST amplification in DF-1 cultures. (**G**) Four different PCRs were used to assess the integrity of the SARS-2-ST gene sequence inserted in the MVA-ST genome. Specifically amplified DNA fragments demonstrated the expected molecular weight of 1.341 kb (specific for S nucleotides 53-1443), 0.714 kb (specific for S nucleotides 1243-1957), 0.954 kb (specific for S nucleotides 1714-2668) and 2.025 kb (specific for S nucleotides 1714-3739) from the SARS-2-ST gene sequence.

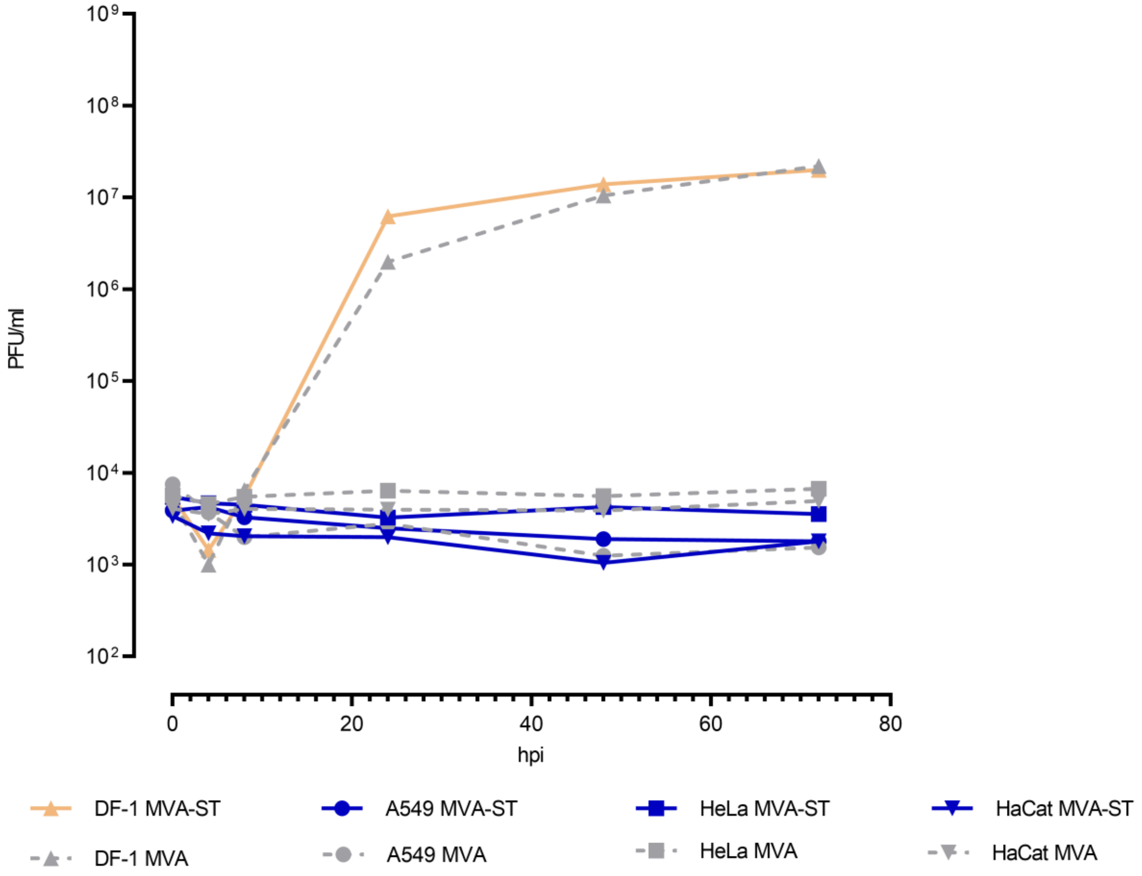

Supplemental Figure 2. Multiple-step growth analysis of recombinant MVA-ST and MVA. Cells were infected at a multiplicity of infection (MOI) of 0.05 with MVA-ST or MVA and collected at the indicated time points. Titration was performed on CEF cells and plaque-forming units (PFU) were determined. MVA-ST and MVA could be efficiently amplified on DF-1 cells but failed to productively grow on cells of human origin (HaCat, HeLa and A549).

A

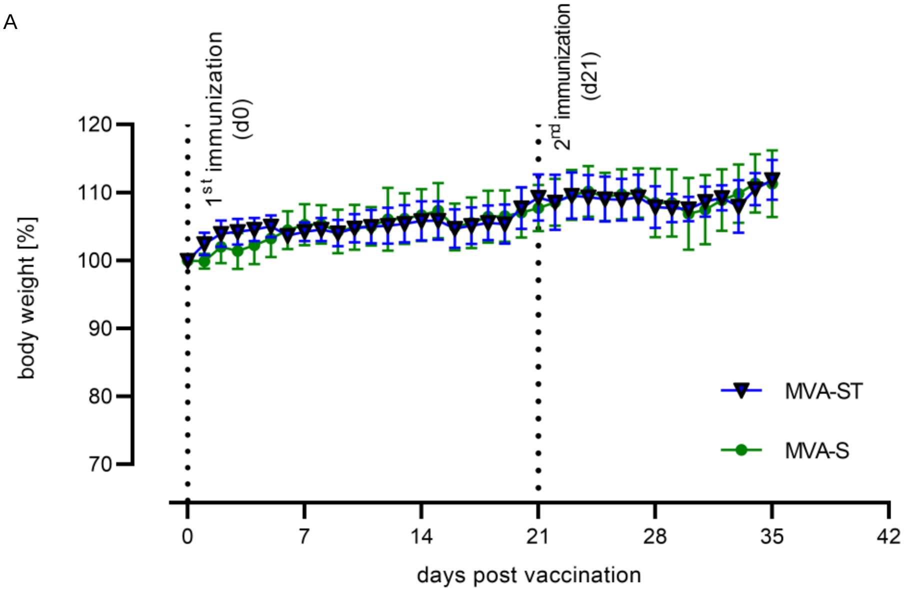

B

Histopathological findings

(summary data)

Main groups | **Sex: female**

**Group**		**1**			**2**			**3**		
**Treatment**		**Vehicle**			**MVA-SARS-2-ST prime boost**			**MVA-SARS-2-S prime boost**		
**Dose**					**10*8 pfu/animal**			**10*8 pfu/animal**		
**Day of necropsy**		**35**			**35**			**35**		
**Grading**		**.**	**1**	**2**	**.**	**1**	**2**	**.**	**1**	**2**
**Organs:**	**Microscopic findings:**									
Adrenal gland	No finding(s)	6/6	0/6	0/6	8/8	0/8	0/8	8/8	0/8	0/8
Kidney, L	No finding(s)	6/6	0/6	0/6	8/8	0/8	0/8	8/8	0/8	0/8
Liver	Lymphoid cell infiltration, periportal, focal	4/6	2/6	0/6	6/8	2/8	0/8	8/8	0/8	0/8
Lungs	Prominent BALT	5/6	1/6	0/6	7/8	1/8	0/8	7/8	1/8	0/8
Ln. subiliacus, L	Lymphoid hyperplasia	3/3	0/3	0/3	0/8	8/8	0/8	0/7	7/7	0/7
Ln. ischiadicus, L	Lymphoid hyperplasia	5/5	0/5	0/5	5/8	3/8	0/8	6/7	1/7	0/7
Ln. popliteus, L	Lymphoid hyperplasia	6/6	0/6	0/6	0/7	7/7	0/7	0/8	8/8	0/8
Ln. iliacus medialis, L	Lymphoid hyperplasia	5/5	0/5	0/5	0/7	7/7	0/7	0/8	8/8	0/8
Thymus	No finding(s)	6/6	0/6	0/6	8/8	0/8	0/8	8/8	0/8	0/8
Spleen	No finding(s)	6/6	0/6	0/6	8/8	0/8	0/8	8/8	0/8	0/8
Site of administration, L	Mixed cell infiltration, interstitial, multifocal	6/6	0/6	0/6	4/8	4/8	0/8	4/8	4/8	0/8
Site of administration, L	Necrosis, myofibres	6/6	0/6	0/6	7/8	1/8	0/8	8/8	0/8	0/8
Site of administration, L	Degeneration, myofibres	4/6	2/6	0/6	0/8	8/8	0/8	0/8	8/8	0/8

No. of animals affected / total No. of animals.

Supplemental Figure 3. MVA-ST and MVA-S immunization and monitoring for side effects. (**A**) Groups of BALB/c mice were vaccinated twice with 10^8 PFU MVA-ST and MVA-S via the intramuscular route using a prime-boost schedule (21-day interval). Shown are body weight changes of mice after prime-boost vaccination with MVA-ST or MVA-S. Body weights was measured daily. No side effects were observed. (**B**) MVA-ST and MVA-S immunization schedules and monitoring for side effects, continuation. Histopathological examinations in prime-boost vaccinated animals. L = Left side. No lesions could be attributed to MVA-ST or MVA-S inoculation in any tissue other than the injection site and draining lymph nodes. Systemic effects related to vaccination were not seen. Signs of minimal to mild myodegeneration at the injection site were observed in treated and control mice. Local inflammation of the myofiber interstitium and the adjacent adipose tissue was observed and interpreted as part of the physiological immune reaction to the vaccine virus as a consequence of the treatment procedure. The degree and extent of inflammation, myodegeneration and necrosis was in accordance with the ratio of inoculum volume in relation to the administration site.

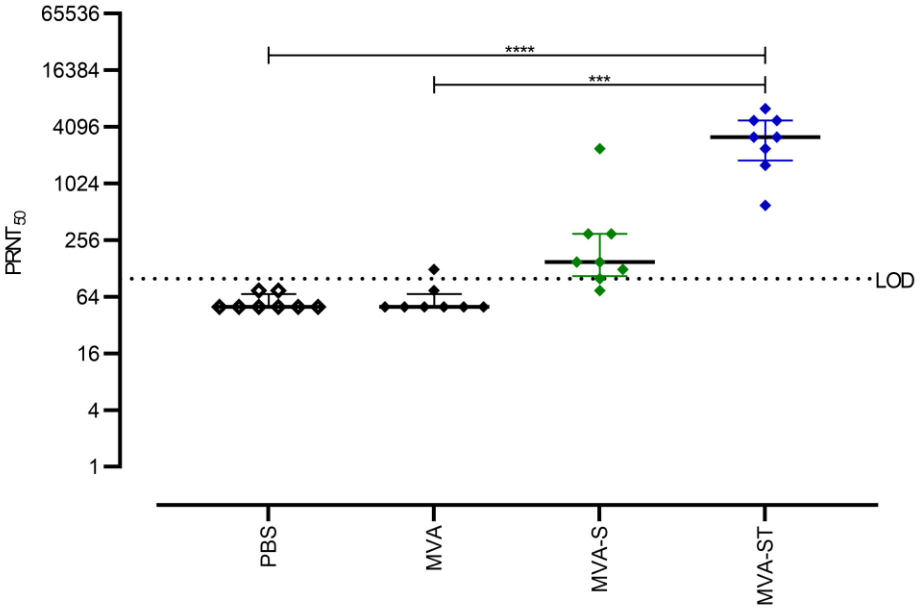

Supplemental Figure 4. Virus-neutralizing antibody responses to SARS-CoV-2 BavPat1 virus strain in vaccinated BALB/c mice. BALB/c mice were i.m. vaccinated in a prime-boost regime (21-day interval) with 10^8 PFU of MVA-S or MVA-ST. Mice inoculated with MVA and saline (PBS) served as controls. Sera were collected 18 days after the first immunization (prime n=10) and 14 days after the second immunization (prime-boost n=10). Sera were analyzed for SARS-CoV-2-S neutralizing antibodies against SARS-CoV-2 BavPat1 virus strain. *** $p < 0.001$, **** $p < 0.0001$. Kruskal-Wallis test and Dunn´s multiple comparisons test. LOD, limit of detection.

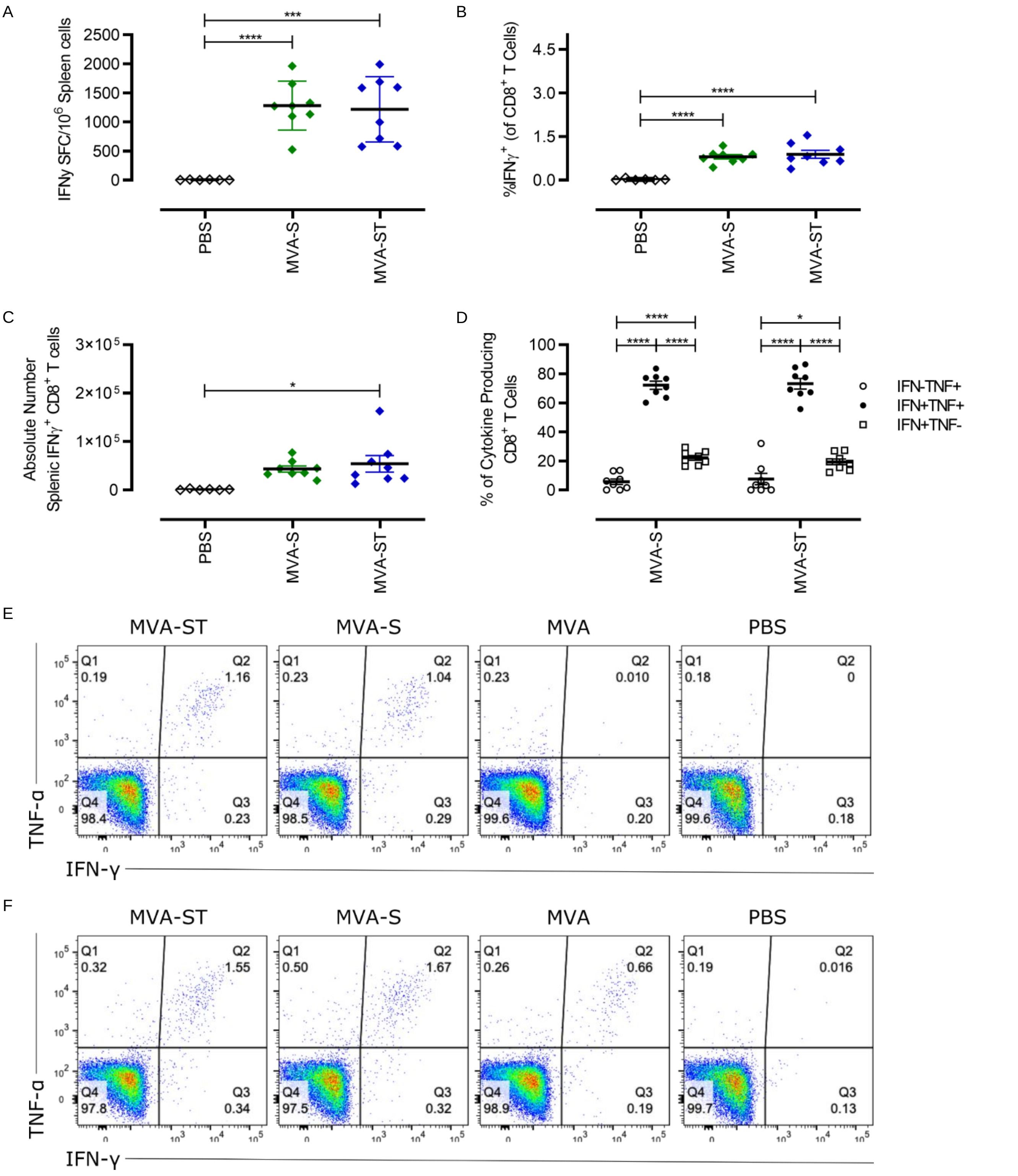

Supplemental Figure 5. Activation of MVA-specific CD8+ T cells after prime-boost immunization (21-day interval) with MVA-ST and MVA-S. Groups of BALB/c mice (n = 6 to 8) were immunized twice with 10^8 PFU MVA-ST and MVA-S over 21-day interval via the i.m. route. Mock immunized mice (PBS) served as controls. Splenocytes were collected and prepared 14 days after the 2nd immunization. Total splenocytes were stimulated with the H2d restricted MVA-specific peptide $F2(G)_{26\text{-}34}$ and measured by IFN-y ELISPOT assay and IFN-y and TNF-α ICS plus FACS analysis. (**A**) IFN-y spot forming colonies (SFC) for stimulated splenocytes measured by ELISPOT assay. (**B** and **C**) IFN-y production by CD8+ T cells measured by FACS analysis. Graphs show the frequency and absolute number of IFN-y+ CD8+ T cells. (**D**) Cytokine profile of $F2(G)_{26\text{-}34}$-specific CD8+ T cells. Graphs show the mean frequency of IFN-γ-TNF-α+, IFN-γ+TNF-α+ and IFN-γ+TNF-α- cells within the cytokine positive CD8 T cell compartment. Representative flow cytometry plots for (**E**) $S_{268\text{-}276}$ and (**F**) $F2_{26\text{-}34}$ stimulated splenocytes. * $p < 0.05$, *** $p < 0.001$, **** $p < 0.0001$. One-way ANOVA and Tukey´s multiple comparisons test.

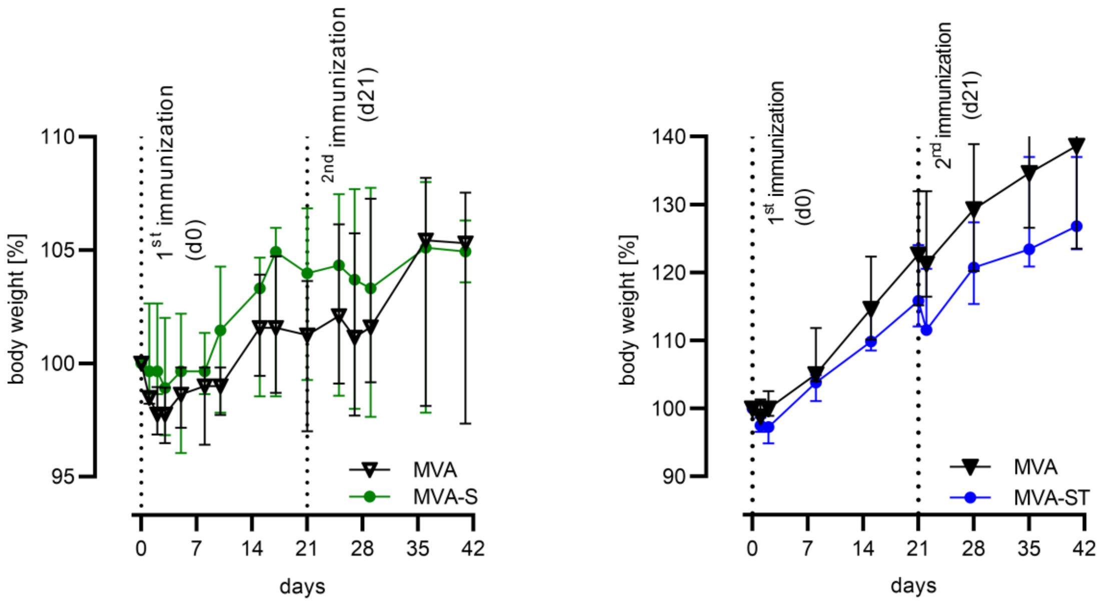

Supplemental Figure 6. MVA-ST and MVA-S immunization and monitoring for side effects. Hamsters were vaccinated with 10^8 PFU MVA-ST and MVA-S via the intra muscular route using a prime-boost schedule (21-day interval). Body weight changes in hamsters after prime-boost vaccination with MVA-ST or MVA-S. Body weight was measured daily. No side effects were observed.

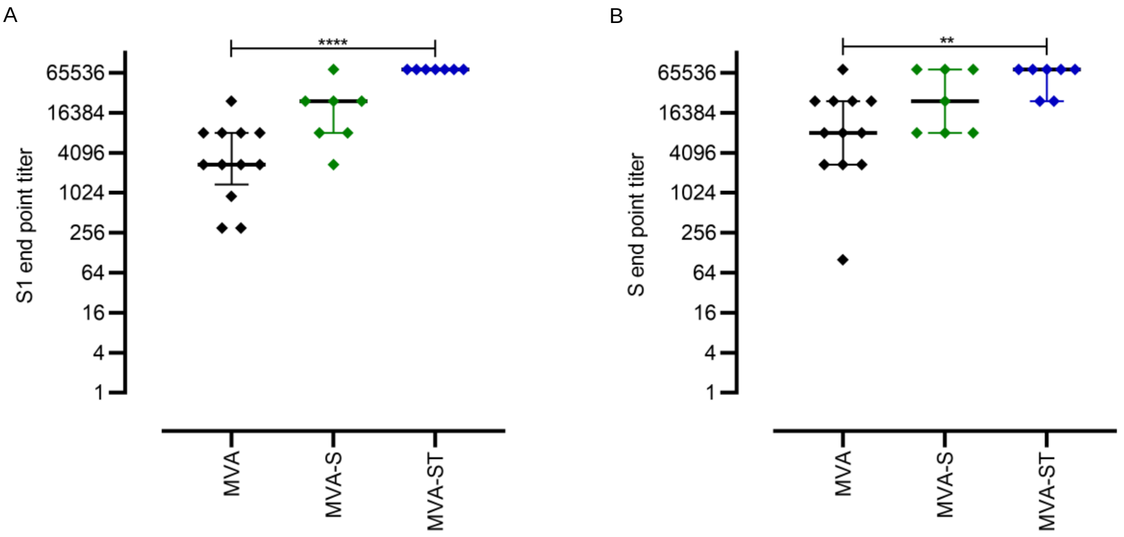

Supplemental Figure 7. Antigen-specific humoral immunity induced in MVA-S or MVA-ST vaccinated hamsters after SARS-CoV-2 BavPat1 challenge infection. Sera collected on day 55 were analyzed for SARS-CoV-S (**A**) S1-specific IgG antibodies and (**B**) full-length S-specific IgG antibodies targeting the BavPat1 SARS-CoV-2 S. ** $p < 0.01$, **** $p < 0.0001$. Kruskal-Wallis test and Dunn´s multiple comparisons test.
